# 50 MHz WSe_2_ Photodetectors from Roll-to-Roll Exfoliated Films

**DOI:** 10.1021/acs.jpclett.6c01579

**Published:** 2026-08-26

**Authors:** Patrick Michel, William Roberts, Yigit Sozen, Andres Castellanos-Gomez, Marcus Scheele

**Affiliations:** † Institute of Physical and Theoretical Chemistry, University of Tübingen, Auf der Morgenstelle 18, 72076 Tübingen, Germany; ‡ 2D Foundry research group, 69570 Instituto de Ciencia de Materiales de Madrid (ICMM-CSIC), Madrid E-28049, Spain

## Abstract

We investigate the time-resolved photodetection characteristics of multilayer WSe_2_ films fabricated via roll-to-roll mechanical exfoliation. By systematically studying the bias and irradiance dependence of the photocurrent dynamics, and by employing spatially resolved photocurrent mapping, we provide insights into the mechanisms governing the device response. We demonstrate a pronounced irradiance dependence of the response times and reveal decay times of 9.8 ns as well as 3 dB electrical bandwidths exceeding 50 MHz, rendering these devices on par with high-quality, manually exfoliated WSe_2_ photodetectors. These results establish roll-to-roll exfoliation as a powerful and scalable fabrication technique capable of producing high-speed photodetectors.

## Graphical abstract

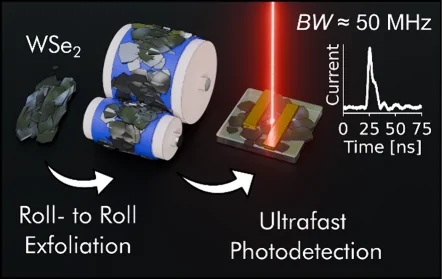

In the search for advanced photodetector materials, few have received as much attention as two-dimensional materials, particularly transition-metal dichalcogenides (TMDCs). They benefit from outstanding optical properties, such as a tunable bandgap^1,2^ and high charge carrier mobilities,^3^ rendering them suitable materials for photodetection in high-speed optoelectronic applications. The main method to obtain high-performing TMDC photodetectors is via mechanical exfoliation, which can be used to produce single crystals with well-defined layer numbers. With this method, remarkable response times down to the nanosecond regime have been achieved. However, the exfoliation of two-dimensional materials is a poorly reproducible process, requiring manual selection of suitable flakes and challenging transfer steps. These requirements make the process hard to automate and pose substantial obstacles to scaling for industrial production. Consequently, alternative methods of the fabrication of varying TMDCs have been investigated, the most well-known methods being chemical vapor deposition (CVD),^4–6^ pulsed laser deposition (PLD)^7–10^ and liquid phase exfoliation (LPE)^11^ which, however, usually exhibit a higher density of defects compared to mechanical exfoliation.^1–4^ Defects are usually detrimental to device speed, reducing charge carrier mobility^12,13^ and introducing deep trap states, preventing fast excitonic recombination and prolonging the photocurrent decay.^14–18^ Therefore, extrinsic response times of photodetectors obtained from scalable methods are typically slower, ranging from seconds to milliseconds, with only few devices reaching microseconds, which is still insufficient for high-speed optoelectronic applications like data transfer. Recently, an all-dry “roll-to-roll” mechanical exfoliation method has been reported to produce large area TMDC films with transfer yields of up to 75%. Using this approach, WSe_2_ films have demonstrated mobilities of up to 3.4 cm^2^/(V s) and responsivities as high as 117 A/W.^19,20^ Preliminary photoresponse measurements showed that photodetection was faster than the 20 ms detection limit of the employed setup.

Here, we present an in-depth study of the time-resolved photoresponse of roll-to-roll exfoliated, multilayered WSe_2_ photodetectors. We analyze the bias- and irradiance-dependence of their photocurrent dynamics and apply photocurrent mapping to rationalize our results. We show that the large area WSe_2_ photodetectors obtained by roll-to-roll exfoliation exhibit response times <10 ns and electrical bandwidths >50 MHz, rendering them competitive with manually exfoliated WSe_2_.

Figure 1a displays a roll-to-roll exfoliated WSe_2_ nanosheet network on a SiO_2_/Si substrate with lithographically defined Cr/Au top contacts. Profilometry shows an average WSe_2_ nanosheet film thickness of ≈100 nm. The Cr/Au (3/60 nm) electrodes were deposited by e-beam evaporation. Additional micrographs, film profilometry and Raman spectra of the material are provided in the Supporting Information (SI) (Figures S1–S3). The channel length and width of this two-terminal WSe_2_ device are 20 and 140 μm, respectively. Consistent with ref 19., the WSe_2_ nanosheet film consists of ≈1–10 μm size flakes with >80% coverage.

**Figure 1:**
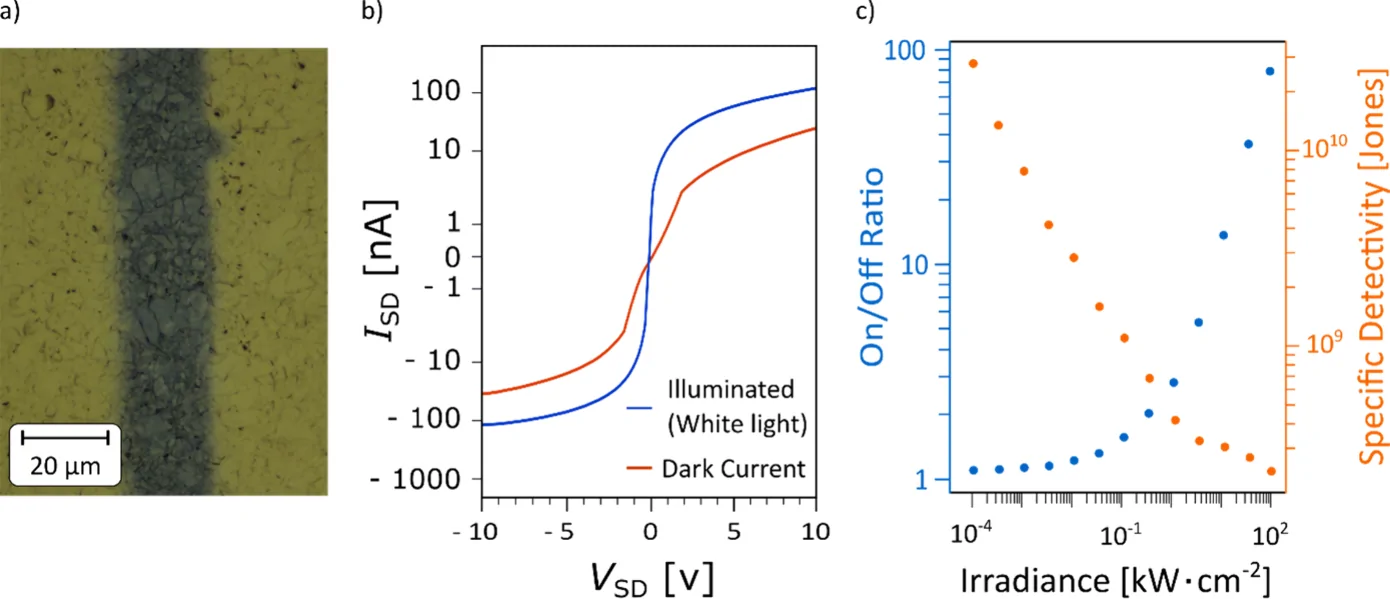
(a) Optical microscopy image of a roll-to-roll exfoliated WSe_2_ film (5 transfers from roll-to-roll) with Cr/Au electrodes evaporated on top. (b) Source-Drain sweeps of the device shown in (a), with a 20 μm × 1 mm channel, in the dark (red) and under approximately 2 × 10^–2^ W/cm^2^ white light illumination (blue). (c) Irradiance dependence of the On/Off ratio (blue) and detectivity (orange) of a 20 μm × 1 mm channel device under 635 nm CW illumination and 1 V bias.

In Figure 1b, we examine the current vs voltage (*I*-*V*) characteristics of the device shown in Figure 1a. We find mostly symmetric, back-to-back diode-like transport characteristics in the dark and under white light illumination (irradiance ≈ 2 × 10^–2^ W/cm^2^). We further report the On/Off ratio and specific detectivity under 635 nm excitation and 1 V bias, visualized in Figure 1c. The detectivity is obtained via

1 $$D^{*}=R\cdot \frac{\sqrt{A}}{\sqrt{2\cdot q\cdot I_{\mathrm{dark}}}}$$

with the responsivity $R=\frac{I_{\mathrm{light}}-I_{\mathrm{dark}}}{P_{\mathrm{opt}}}$ , area *A*, the elemental charge *q*, the current in the dark or under illumination *I*_dark_/*I*_light_, and the optical power *P*_opt_. To a first-order approximation, we assume *I*_dark_ to be the dominant noise contribution.^21^ This method however can lead to an overestimation of the detectivity, if a different noise type is predominant. To address this problem, a consensus statement by several authors of the photodetector community^22^ has proposed to report the specific detectivity in a standardized manner, using

2 $$D^{*}=D\sqrt{A\cdot B}$$

Herein *A* is the illuminated channel area and *B* is 1.57 × *f*_3 dB_ if a first-order low-pass filter characteristic is assumed. The detectivity *D* is defined by *D* = 1/*NEP*, where *NEP* is the noise equivalent power, at which the signal-to-noise-ratio (SNR) is *P*_signal_/*P*_noise_ = 1. By extrapolation from the performed measurements, we estimate a *NEP* of 53 nW, resulting in a D* of 1.5 × 10^7^ Jones. For further information on the methodology, we refer to the Supporting Information (Figure S4).

Varying the irradiance from 10^–4^ to 10^2^ W/cm^2^ results in the common increase of the On/Off ratio at the expense of a lower detectivity, reflecting the general trend toward photosaturation. For details regarding the data acquisition, irradiance-dependence of photocurrent, responsivity and external quantum yield (EQE) as well as spectrally resolved responsivity, the reader is referred to the Supporting Information (Figures S5–S7).

In Figure 2a we display the time-resolved photoresponse measurements with 635 nm light pulses, revealing bias-dependent fall times between 10 and 22 μs. The complete data set, including rise- and fall flanks, is provided in the Supporting Information (Figure S8a). In Figure 2b, higher irradiance yields faster photoresponse, changing by approximately 2 orders of magnitude over the same range of irradiance. The irradiance-dependent measurements are consistent with earlier reports showing that the response time in TMDC photodetectors is typically RC-limited,^23^ as we observe shorter fall times at higher irradiances. For irradiances ≥10 kW/cm^2^, we need to increase the repetition rate to 100 kHz to fully resolve the response dynamics. Comparative photocurrent measurements are provided in the Supporting Information (Figure S8b). The saturation of the photocurrent was verified for a comparative device at repetition rates of 100 Hz up to 500 kHz (see Figure S9 and Table S1). A detailed summary of the results depicted in Figure 2b is provided in the Supporting Information (Tables S2 and S3).

**Figure 2:**
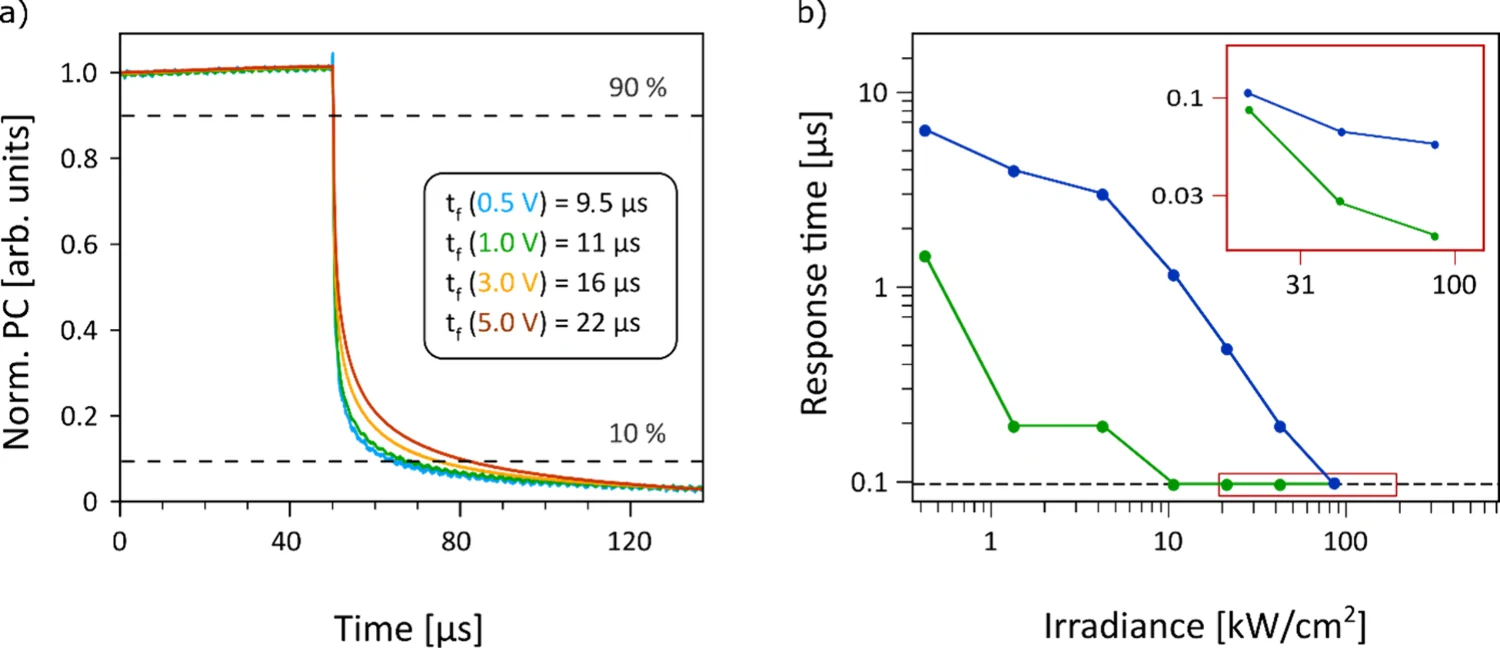
(a) Transient photocurrent response (at the fall segment) of a 25 μm **×** 1 mm channel device under 635 nm square-pulse excitation (1 kHz repetition rate, irradiance ≈2 × 10^–3^ W/cm^2^) at different bias voltages. The 10–90% thresholds used to extract the fall time are indicated by dashed lines. (b) Irradiance dependence of the rise (green) and fall times (blue) for a 40 μm × 1 mm channel at 3 V bias under 635 nm excitation and 10 kHz repetition rate, with an inset showing additional measurements conducted at a 100 kHz. The dashed horizontal line at ∼100 ns indicates the instrumental resolution limit at 10 kHz. Measurements in (a) and (b) were performed on different optical setups (probe station vs confocal microscope), resulting in differing power densities. Experimental details are provided in the SI.

No correlation between channel length and photoresponse has been observed for measurements with channel lengths between 5 and 80 μm. An overview of the temperature- and channel-length-dependent measurements can be found in the Supporting Information (Figures S10 and S11).

To further investigate the apparent lack of channel-length dependence and the counterintuitive increase of the fall time with increasing bias (Figure 2a), we perform photocurrent mapping (Figure 3). A linear-scale representation of the absolute photocurrents is provided in the Supporting Information (Figure S12). For small biases (±0.1 V), we find that the majority of the photocurrent is generated in close proximity to the metal contacts. We note a significant photocurrent even at zero bias, suggesting a Schottky-type contact (in good agreement with the observed back-to-back diode-like *I–V* characteristics). While certain hotspots and an uneven distribution of generated photocarriers along the metal/WSe_2_ interface are evident, there is no obvious correlation with the flake-to-flake boundaries in the film which we display in terms of its scattering image from Figure 1a. At higher fields (±1 V), the photocurrent generation extends over the entire channel. Under such high field conditions, a significant number of photocarriers generated far away from the metal contacts can be efficiently collected, diffusing across multiple flake-to-flake boundaries and long percolative paths. We suggest that this may be the origin of the longer fall times at large biases in Figure 2a. Additional photocurrent mapping performed on a second device is presented in the Supporting Information (Figure S13), demonstrating comparable field-dependent behavior.

**Figure 3:**
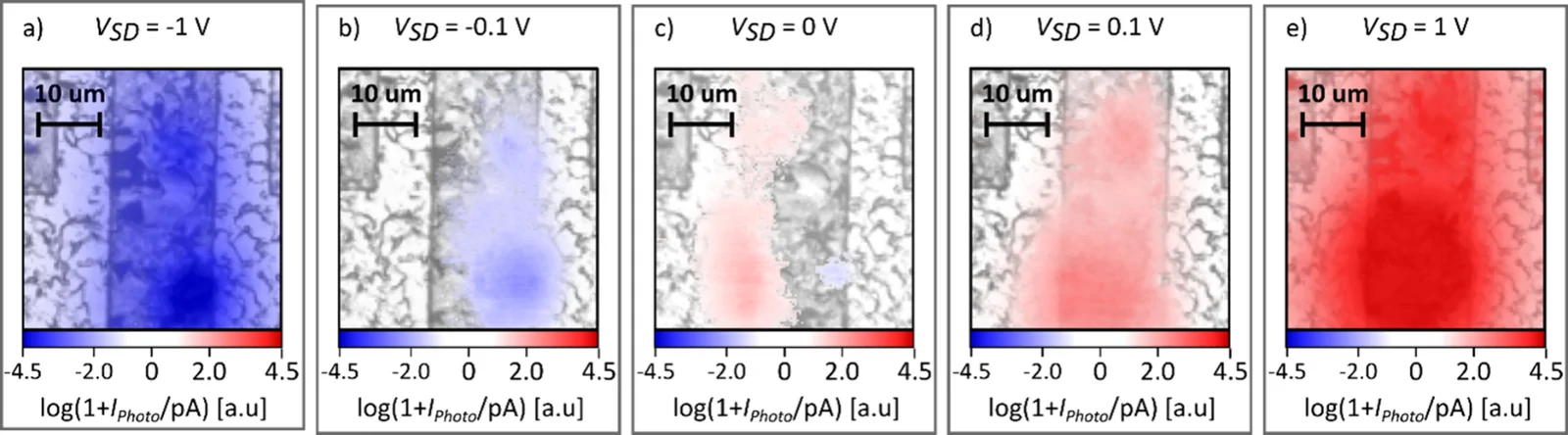
Scattering image of the channel depicted in Figure 1a with overlaid photocurrent maps for several bias-voltages. To enable uniform scaling across all subfigures, a log­(1 + *I*_Photo_/pA) function is applied to the current values. Measurements were performed with a wavelength of 532 nm (*P*_opt,532 nm_ ≈ 1.4 mW at 78 MHz repetition rate) chopped to a 700 Hz frequency.

To assess the potential of roll-to-roll exfoliated WSe_2_ for fast photonic data transfer, we analyze the fall time after a ∼ 50 ps laser impulse in Figure 4a. From the time-dependent photocurrent *I*(*t*) recorded during this measurement, we obtain the power spectrum via Fourier-transformation according to *P*(ω) = |FFT­(*I*(*t*))|^2^ which affords the electrical bandwidth at 3 dB attenuation (*f*_3 dB_). An exemplifying measurement exhibits a fall time of 9.8 ns and yields a 3 dB cutoff frequency of *f*_3 dB_ = 53.5 MHz. Measurements demonstrating a complete decay of the impulse photoresponse for pulse repetition rates between 150 kHz and 2 MHz are provided in Figure S14 and Table S4. A schematic of the experimental setup (Figure S15), along with a table summarizing the individual electrical components and their respective speed limitations, is provided in Table S5. The reproducibility of the high-speed switching was verified for five additional devices, and a statistical overview of the results is depicted in Figures S16 and S17. For all of these data sets, we refer to the Supporting Information.

**Figure 4:**
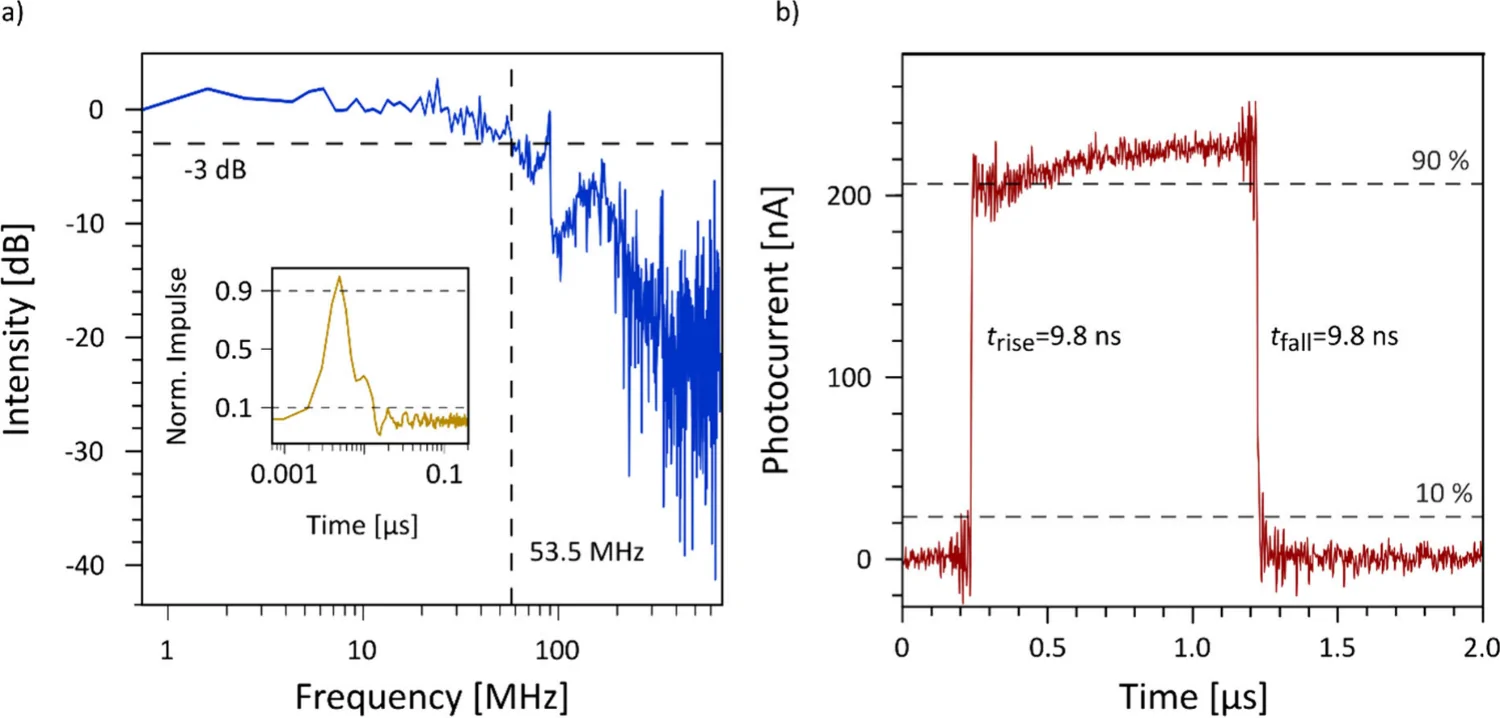
Time-resolved photocurrent measurements of a 30 μm × 1 mm channel. (a) Power spectrum of the device, obtained via Fourier transformation of the impulse photoresponse shown in the inset. The measurement was performed with a 636 nm impulse laser at 1 MHz repetition rate under a 1 V bias. The 3 dB bandwidth (53.5 MHz) is indicated by the dashed lines. The average irradiance is **≈**2 × 10^3^ W/cm^2^. (b) Square-pulse measurement conducted with a 635 nm square pulse illumination (665 μW focused to approximately 1 μm spot diameter, irradiance **≈**8 × 10^4^ W/cm^2^) at a repetition rate of 500 kHz and 3 V bias voltage. The 10% and 90% thresholds are shown with dashed lines.

## Discussion

We contextualize our results by comparison with other large-scale fabrication approaches, such as CVD and LPE, as well as manually exfoliated (small-area) devices in Figure 5. This overview highlights that roll-to-roll mechanically exfoliated WSe_2_ affords response times that are competitive even with manually exfoliated devices (made from single-crystal flakes), however with the unique advantage of rapid, semiautomatic, and scalable device production up to the wafer scale. We acknowledge that this method produces bulk-like multilayers, while manual exfoliation often focuses on mono/few-layer devices to take advantage of a direct bandgap, lower out-of-plane drift and reduced interlayer diffusion. In practice, however, bulk TMDCs have also been shown to offer distinct advantages for fast photodetection, such as a reduced influence of surface defects, higher absorption rates and higher charge carrier mobilities.^24,25^ This is reflected in the responsivity, detectivity, response times, and *f*_3 dB_ values reported here, which compare favorably with the large body of TMDC-photodetector literature.^4–11,21,26–55^

**Figure 5:**
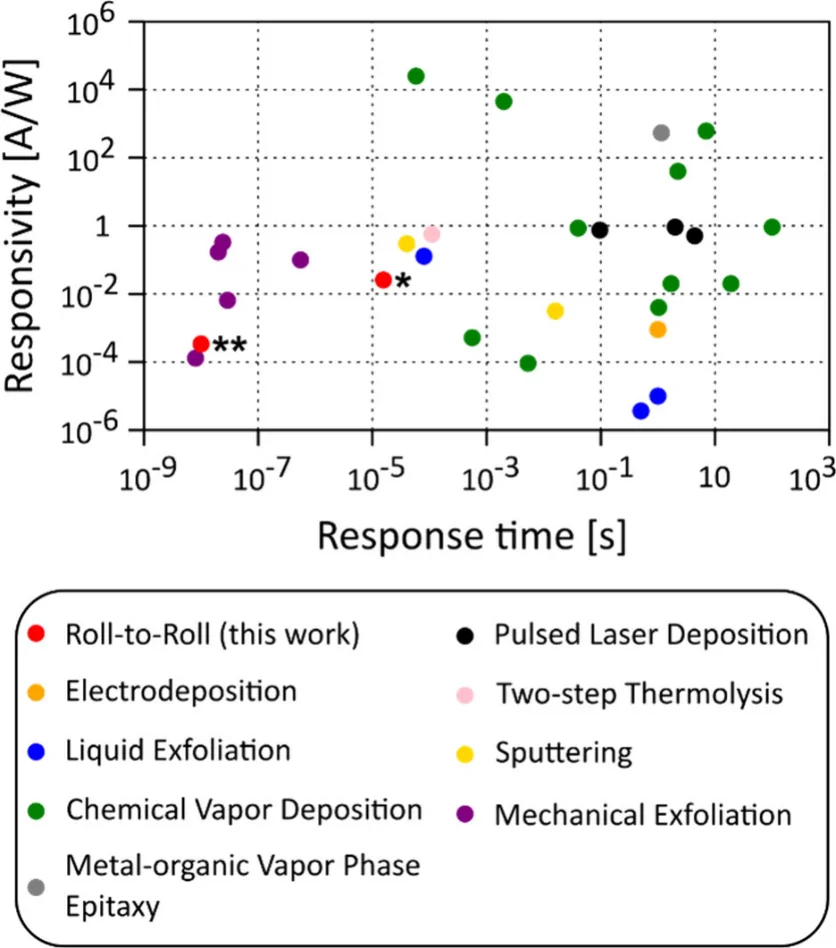
Comparison of responsivity and response time between roll-to-roll exfoliated devices and other large-scale fabrication methods for TMDCs. Herein, the slowest reported rise- or fall time was taken as the response time, as this was considered the limiting factor for the device. In this representation, devices from different TMDC materials are included. A detailed literature review, including details on the materials, is presented in the Supporting Information (Table S6).^4–11,21,26–55^ The responsivity values for this work (roll-to roll) were calculated from Figure 2a (*) and Figure 4b (**). The applied irradiances differ considerably (2 × 10^–3^ W/cm^2^ and 8 × 10^4^ W/cm^2^, for Figure 2a and Figure 4b, respectively), demonstrating a trade-off between the measured device speed and responsivity.

An inherent draw-back of roll-to-roll exfoliation is the inhomogeneous, interconnected flake network nature of the TMDC film, which—as we suggest—invokes the negative correlation between bias voltage and response time. In a homogeneous photodetector, increasing the bias voltage typically results in faster rise- and fall times. Future fabrication refinements should therefore focus on even more homogeneous films with larger grain sizes. Furthermore, the apparent Schottky-nature of the metal/WSe_2_ interface revealed by *I*-*V* characteristics (Figure 1b) and photocurrent mapping (Figure 3) could be improved.

The pronounced correlation of response time with irradiance (Figure 2b) is often attributed to a decrease in photoresistance at higher irradiances, which reduces the RC time^47^ and leads to a trade-off between response speed and responsivity or photogain.^56^ This highlights the importance of providing intensity/irradiance-dependent data for meaningful comparisons between different materials. While the large irradiances used here to achieve the exceptionally fast response times in Figure 2b may be impractical for certain applications, we stress that this is only necessary to maintain the full photocurrent intensity. At lower irradiances, a significant portion of the photocurrent still decays at these fast time scales (cf. Figure S18), in addition to a slow component, for instance due to RC limitation. Consequently, the fast response times are largely retained at the expense of a photocurrent signal reduced to approximately 70%, which may still be sufficient for some applications.

Our measurements show a sublinear correlation between the response times and responsivity whitin the applied irradiances, observable in Figure 1c) and in the SI (Table S2). This behavior is consistent with earlier reports,^57^ and attributable to faster recombination rates at higher exciton densities^58–60^ as well as a variety of further effects.^18,60,61^ Our measurements also operate at high optical powers, where photogating, an explanation for high responsivities at low optical irradiances, is suppressed and response are speeds governed mainly by RC-time reductions.^62,63^

In conclusion, we report decay times of 9.8 ns and 3 dB-bandwidths of ≈50 MHz for WSe_2_ photodetectors obtained by roll-to-roll mechanical exfoliated, all-dry transfer.^20^ We observe a strong irradiance dependence of the photocurrent dynamics and an inverse correlation between the applied bias and the speed of photodetection. We attribute the irradiance dependence to a decrease in photo resistance, and the bias dependence to an inhomogeneous film composition that leads to localized hotspots of charge carrier generation. Our results show that roll-to-roll exfoliation is a promising, automatization-suitable method for fabricating large-area TMDC photodetectors with high-speed photoresponse, which can be further optimized to address the intrinsic trade-off between responsivity/detectivity^56^ and speed.

## Supplementary Material



## Notes

Methods and materials, optical micrographs, film profilometry, Raman characterization, irradiance-dependent photocurrent measurements, photocurrent spectroscopy, bias- and irradiance dependent time-resolved measurements, comparative time-resolved data at different laser repetition rates, temperature-dependent rise- and fall times, channel-length dependent rise- and fall times, additional material regarding scanning photocurrent microscopy, tabular literature review regarding time-resolved properties of large-scale TMDC photodetectors (PDF)
