# Supplementary material for: 50 MHz WSe2 Photodetectors from Roll-to-Roll Exfoliated Films
Source: J Phys Chem Lett. 2026 Aug 26;17(36):10413–9. doi: 10.1021/acs.jpclett.6c01579 (PMC13573613; doi:10.1021/acs.jpclett.6c01579)
Supplement: Supplementary file 1 [file jz6c01579_si_001.pdf]

Supporting Information:

50 MHz WSe_2 Photodetectors from Roll-to-Roll Exfoliated Films

Patrick Michel[a#], William Roberts[a#], Yigit Sozen[b], Andres Castellanos-Gomez[,b], Marcus Scheele[*,a]*

[a]Institute of Physical and Theoretical Chemistry, University of Tübingen, Auf der Morgenstelle 18, 72076 Tübingen, Germany

[b]2D Foundry research group, Instituto de Ciencia de Materiales de Madrid (ICMM-CSIC), Madrid E-28049, Spain

[#] These authors contributed equally.

*Corresponding Authors: Marcus Scheele, Email: marcus.scheele@uni-tuebingen.de; Andres Castellanos-Gomez, E-mail: andres.castellanos@csic.es

Methods and Materials

Roll-to-roll Mechanical Exfoliation

Roll-to-roll mechanical exfoliation[1] was performed using two polyoxymethylene (POM) cylinders with an incommensurate diameter ratio of 53:23. Through this geometric mismatch, the cylinders require 1219 complete revolutions to return to their initial relative alignment, which ensures uniform distribution of exfoliated material across the adhesive surfaces. First, each cylinder was wrapped in double-sided Nitto SPV 224 tape, and a bulk WSe_2 crystal (HQ Graphene) was transferred onto the larger-diameter cylinder. Exfoliation was initiated by bringing the cylinders into contact and rotating them for 60 s. The cylinder-rotation was driven by an electric drill coupled to the larger cylinder, operating at approximately 1500 rpm. During the exfoliation process, the cylinders were held together with a constant applied force of ≈ 20-40 N to maintain effective mechanical contact.

Transfer from the Nitto-tape onto the acceptor substrate was performed on a hot plate at 110 °C for 5 minutes. This thermal treatment resulted in a highly efficient transfer process, with the majority of flakes successfully adhering to the acceptor surface.

Photolithography

Fabrication procedure of devices for time-resolved characterization (e.g. **Fig. S1a,b**): The electrode array was fabricated using a SmartPrint maskless lithography system via a double-resist process. First, LOR 2A was spin-coated onto the substrate at 3000 rpm for 40 s and subsequently baked on a hot plate at 180 °C for 3 min. A second resist layer consisting of AZ1505 positive

photoresist was then deposited by spin-coating at 4000 rpm for 40 s, followed by a soft bake at 100 °C for 1 min. The predefined electrode pattern was then exposed for 0.6 s using the 10× microscope objective of the lithography system. After exposure, the resist stack was developed in the developer (AZ 726 MIF) for 19 s, and the samples were rinsed thoroughly with DI water. Afterwards, metal deposition was performed via thermal evaporation.

For scanning photocurrent microscopy (e.g. **Fig. S1c,d**), glass substrates with a thickness of approx. 160 µm were used as substrates and precleaned sequentially with acetone and isopropanol. After WSe_2 deposition, 100 µL of a positive photoresist (ma-P 1205, Microresist Technology) was spin-coated at 3000 rpm for 30 s and soft-baked on a hot plate at 100 °C for 1 min. The electrode pattern was defined by aligning the samples with a photomask and exposing them to UV light using a maskless aligner (Heidelberg Instruments µMLA). Development was carried out by immersing the exposed substrates in ma-D 331/S developer (Microresist Technology) for approximately 30 seconds, followed by two rinsing steps with ultrapure water (Milli-Q). Metal contacts were subsequently formed by thermal evaporation of a 4 nm Chrom layer and a 60 nm gold layer. The lift-off process was performed by immersing the samples in acetone for a few minutes, after which the substrates were rinsed with isopropanol and dried in a nitrogen stream.

Optoelectronic Characterization

Dark-current sweeps and measurements under white-light illumination depicted in **Fig. 1b** were conducted in a Lake Shore Cryotronics CRX-6.5K probe station, where the samples were contacted using gold-coated tungsten tips and measured with a Keithley 2636B source meter. The white light lamp provides an irradiance of approximately 2×10^{-2} W/cm^2. Furthermore, time-

resolved photocurrent measurements were carried out in the probe station for the bias-, temperature- and channel length-dependent measurements (**Fig. 2a**, **Fig. S10** and **Fig. S11**, respectively). Square-pulse excitation was generated using a 635 nm CW laser ($P_{opt} \approx 28$ µW), which illuminates a spot diameter of approximately 1.5 mm. The laser is driven by an FSL500 PicoQuant laser driver and triggered by a Hewlett-Packard 33120A arbitrary waveform generator. In time resolved measurements, photocurrents were first preamplified with a FEMTO DHPCA-100 amplifier (200 MHz bandwidth at 100 V/A gain) and detected via a 600 MHz Zürich Instruments UHF lock-in amplifier, averaged over 4 G to 69 G samples, and subsequently corrected for background contributions.

Focused optical excitation was performed using a custom-built confocal microscopy setup, by focusing the laser beam onto the active device channels through a Zeiss LD Plan-Neofluar 63×/0.75 objective to a spot size of approximately 1 µm. A schematic overview of the setup is provided in **Figure S15**. The speed-limiting specifications of the electrical and optical components are summarized in **Table S5**. Impulse measurements (**Fig. 4a**) were performed with an NKT SuperK FIU-15 PP impulse laser in combination with an NKT tunable high-contrast single-line filter, selecting a wavelength of 636 nm (averaged $P_{opt,636nm} \approx 12$ µW at 1 MHz repetition rate). Square pulse measurements (**Fig. 2b**, **Fig. 4b**) were performed with the 635 nm CW FSL500 PicoQuant laser mentioned above, generating an optical output of $P_{opt} \approx 665$ µW.

The irradiance-dependent measurements depicted in **Fig. 1c** were performed with the same setup, however, the laser (635 nm CW, $P_{opt} \approx 2.3$ mW) was focused to a spot size of approx. 50 µm. All provided power values for focused laser illumination are based on the assumption of a 65 % intensity loss in the beam path due to a (50:50) beamsplitter and other optical elements. The

samples were contacted using tungsten tips (micromanipulators 448000-S-NA) connected via 60 cm RG178 B/U coaxial cables. Non-time-resolved photocurrents were recorded using a Keithley 2634B source meter. For time-resolved experiments, the photocurrent signal was preamplified using a FEMTO DHPCA-100 amplifier. The amplified signals were then recorded with a Zürich instruments 600 MHz UHFLI.

Optical characterization

Spectroscopic and microscopic characterization was performed using the confocal laser setup described above. Raman spectra were acquired by using a 532 nm laser for excitation combined with two razor-edge 532 nm long pass filters in the detection path. Spectroscopic measurements were performed by using an Andor Shamrock 303i-B spectrometer coupled with an Andor iDus 401 CCD camera. For imaging purposes, an avalanche photodiode was used for detection.

Scanning Photocurrent Microscopy (SPCM)

For SPCM measurements (**Fig. 3**, **Fig. S12-S13**), the devices were transferred to a custom PCB chip-carrier and contacted via wire bonding. Laser scanning was performed at the confocal setup described above with a 532 nm impulse laser ($P_{opt,532nm} \approx 1.4$ mW at 78 MHz repetition rate) chopped to a 700 Hz frequency. For detection of the resulting photocurrent, a lock-in amplifier (SRS SR830) was connected to the scanning system. Bias voltages were applied through the auxiliary output of the SR830.

Supplementary Figures

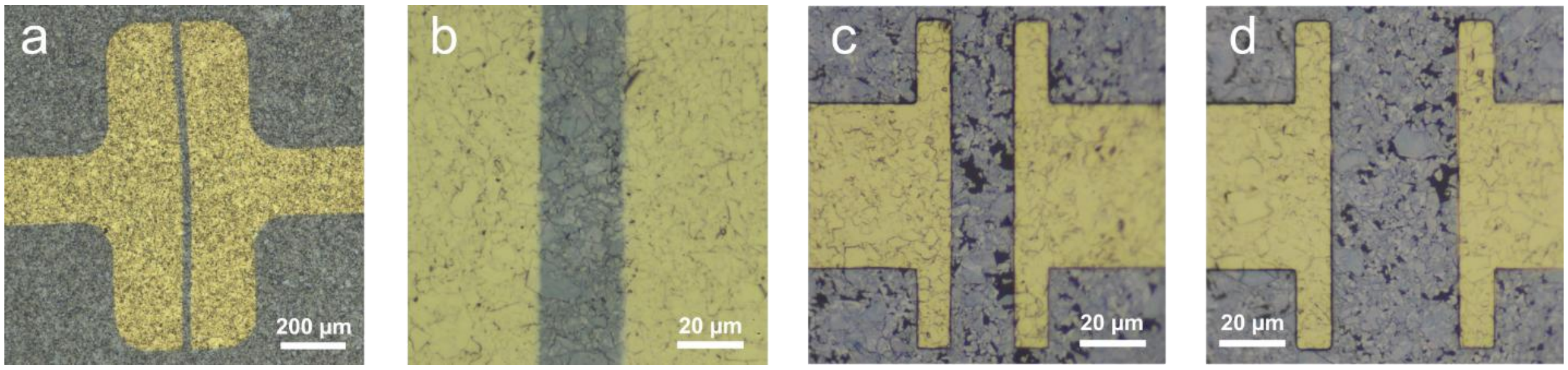

Figure S1. Optical micrograph of exemplary photodetectors fabricated by roll-to roll exfoliation and subsequent metal evaporation. **a)** Micrograph of an exemplary device with 1 mm channel width and 30 µm channel length. **b)** Magnified section of the channel depicted in a). **c)** Device 1 used for photocurrent imaging (**Fig. 3, Fig. S12**), 20 µm channel length and 100 µm channel width. **d)** Device 2 used for photocurrent imaging (**Fig. S13**), 40 µm channel length and 100 µm channel width.

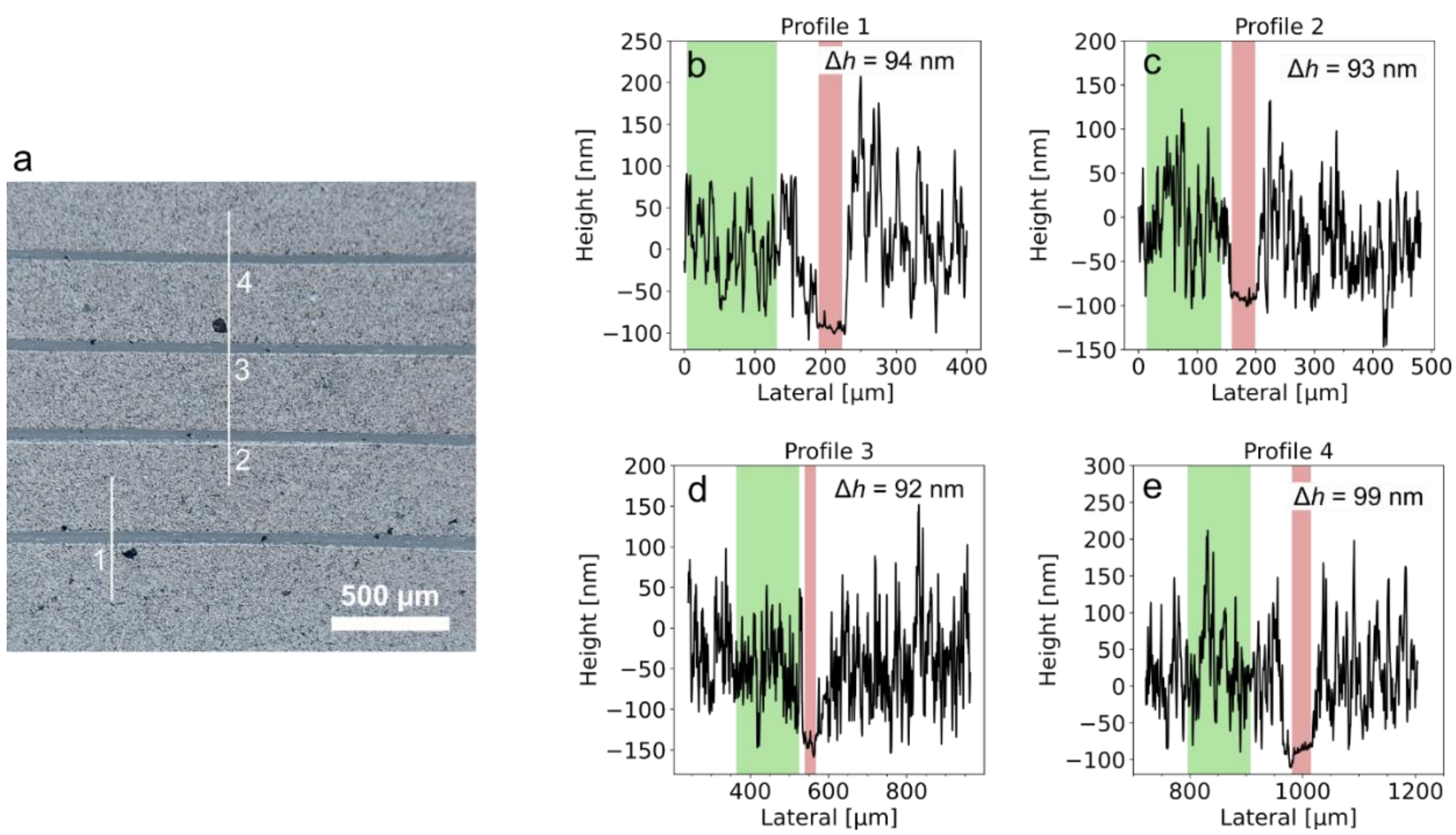

Figure S2. Profilometry investigations of the WSe_2 film depicted in **Fig. S1c,d**, measured with a Bruker Dektak XT-A profilometer. **a)** Optical micrograph of four intentional scratches. **b-e)** Slope-corrected height profiles of scratches 1-4 depicted in a). Since the irregular surface of the films

yields rather irregular profiles, height differences were calculated from mean values of the regions indicated in green and red for film and substrate surface, respectively. The determined heights of scratch 1-4 are all within a 90-100 nm range, which is in good agreement with the data reported by Sozen et al.[2]

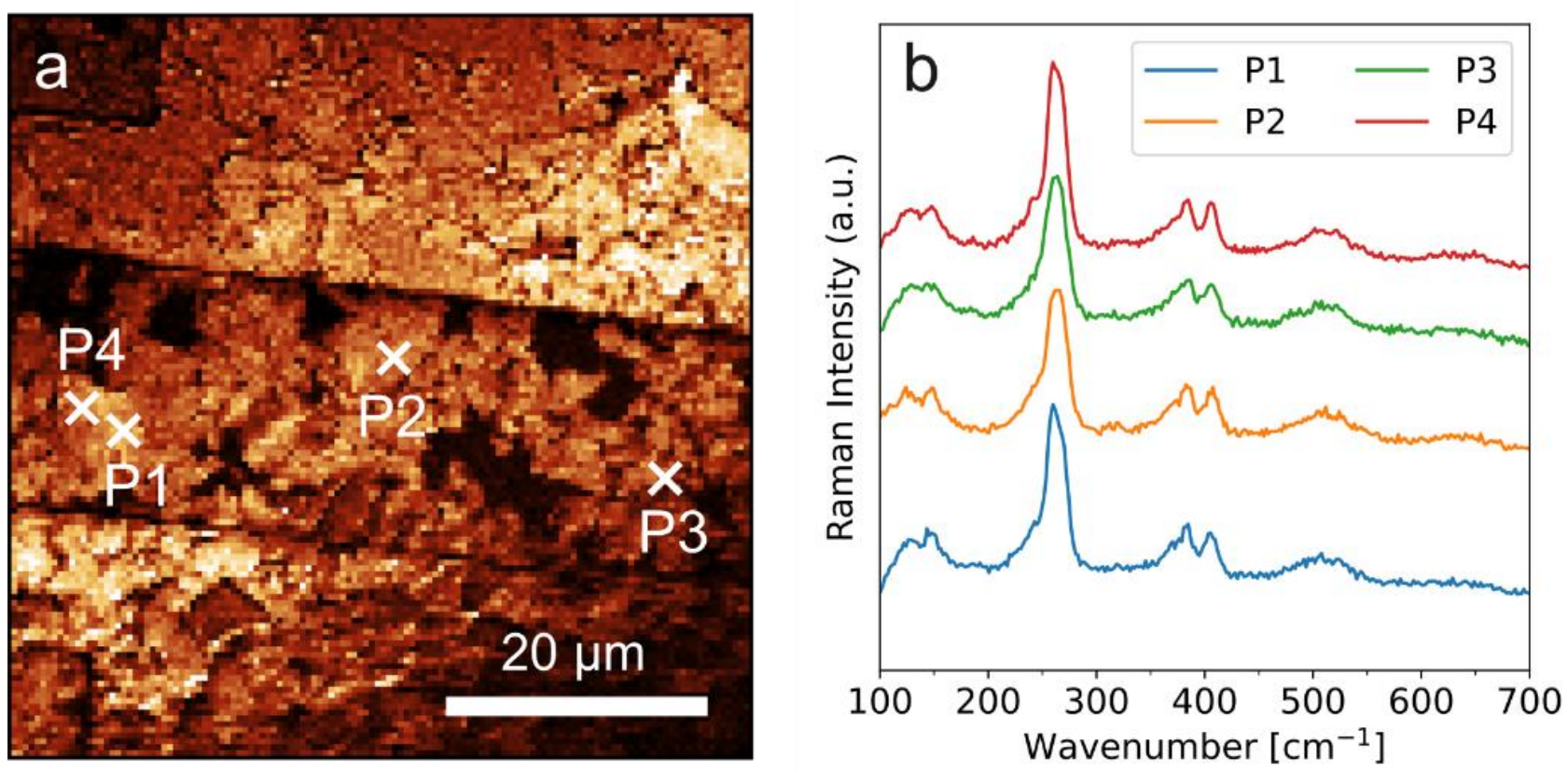

Figure S3. Raman characterization of the WSe_2 film depicted in **Fig. S1c**. **a)** Scanning laser micrograph (scattering mode) with marked positions for Raman characterization. **b)** Raman spectra for the positions marked in a). The spectra are nearly identical for each position and in good agreement with literature reports on bulk WSe_2.[3]

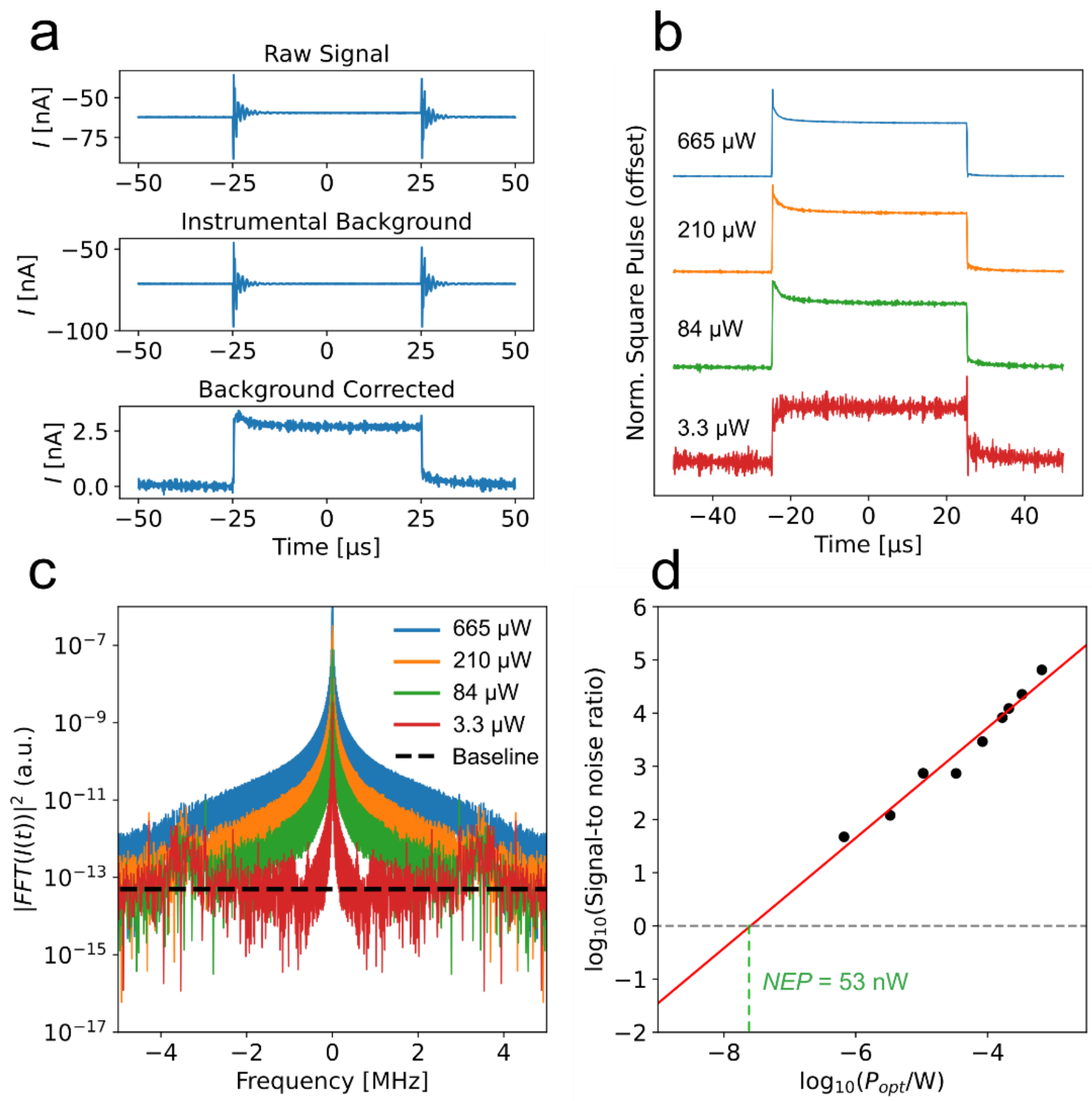

Figure S4. Processing of time-resolved data and extraction of the noise-equivalent power (NEP). A 635 nm CW laser ($P_{\text{opt},0} \approx 665$ µW, spot diameter approx. 1 µm) was modulated with a 10 kHz square pulse, and the resulting photocurrents were detected as described in the Methods section. The channel dimensions were 40 µm in length and 1 mm in width, and the device was operated under a 3 V bias. **a)** Instrumental distortions, recorded in the dark at 0 V bias, were subtracted from the raw signal, resulting in a background-corrected response. **b)** Normalized photoresponse to the square-pulse illumination, showing increasing noise contributions with decreasing optical power. **c)** To extract the signal-to noise ratio (SNR) from the measurements depicted in b), the power spectrum $P(f) \propto |\text{FFT}(I(t))|^2$ is computed and the total power calculated by $\int_{-5\text{ MHz}}^{5\text{ MHz}} |\text{FFT}(I(t))|^2 df$. To separate the signal from underlying noise, an approximate baseline is

introduced, which represents the noise contribution. **d)** The NEP is estimated by extrapolating the linear regression to a SNR of 1.

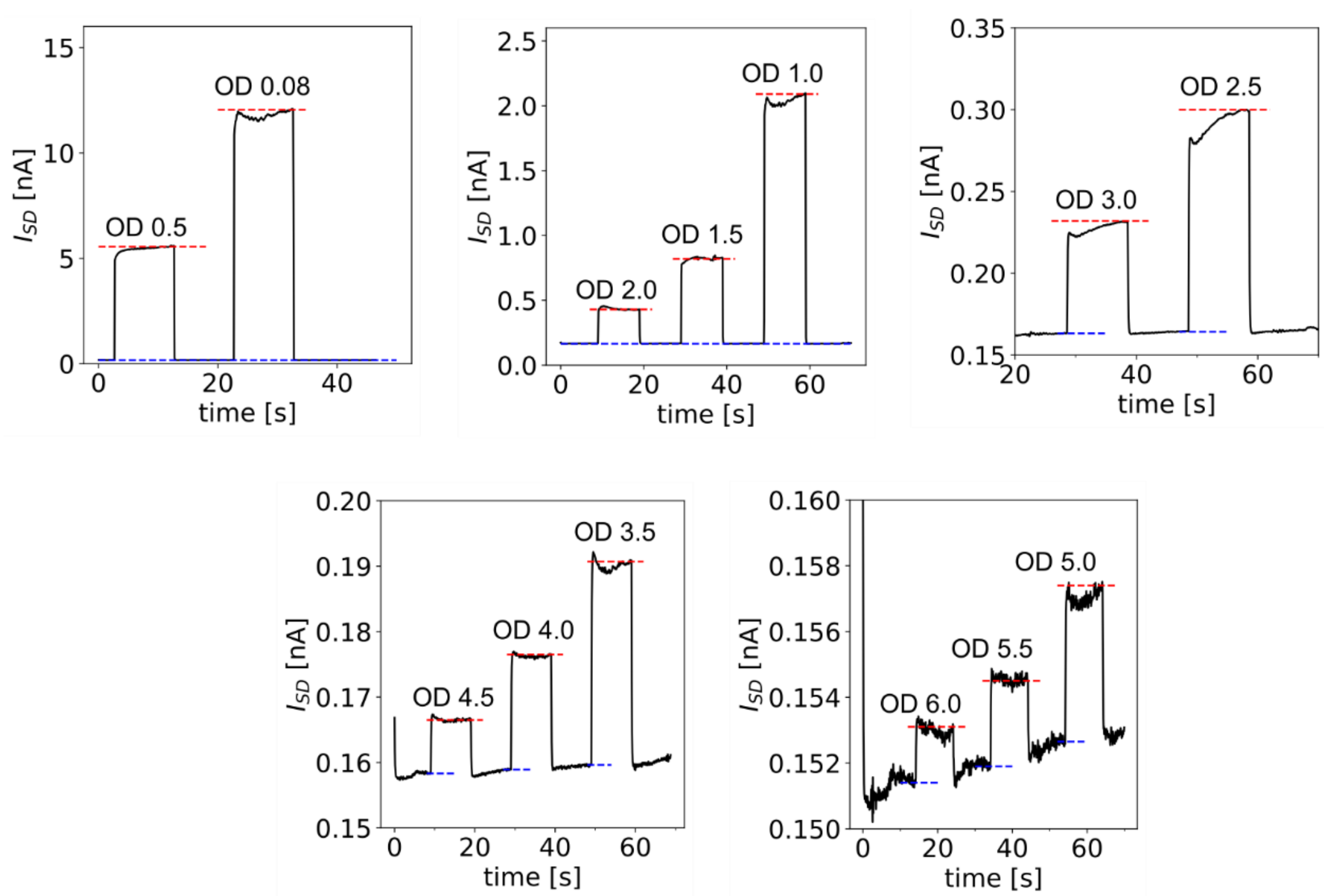

Figure S5. Intensity-dependent photocurrent measurements of one exemplary 20 µm × 1 mm channel under a bias voltage of 1 V. The unattenuated 635 nm CW laser yields an irradiance of approx. 118 W/cm^2 (2.3 mW focused on approx. 50 µm spot diameter). Dark currents and currents under illumination are depicted in blue and red, respectively. In low current measurements, a drift of the baseline becomes apparent. This is corrected by determining individual dark current levels for each pulse. From these measurements, we calculate the data depicted in **Fig. 1c** and **Fig. S6**.

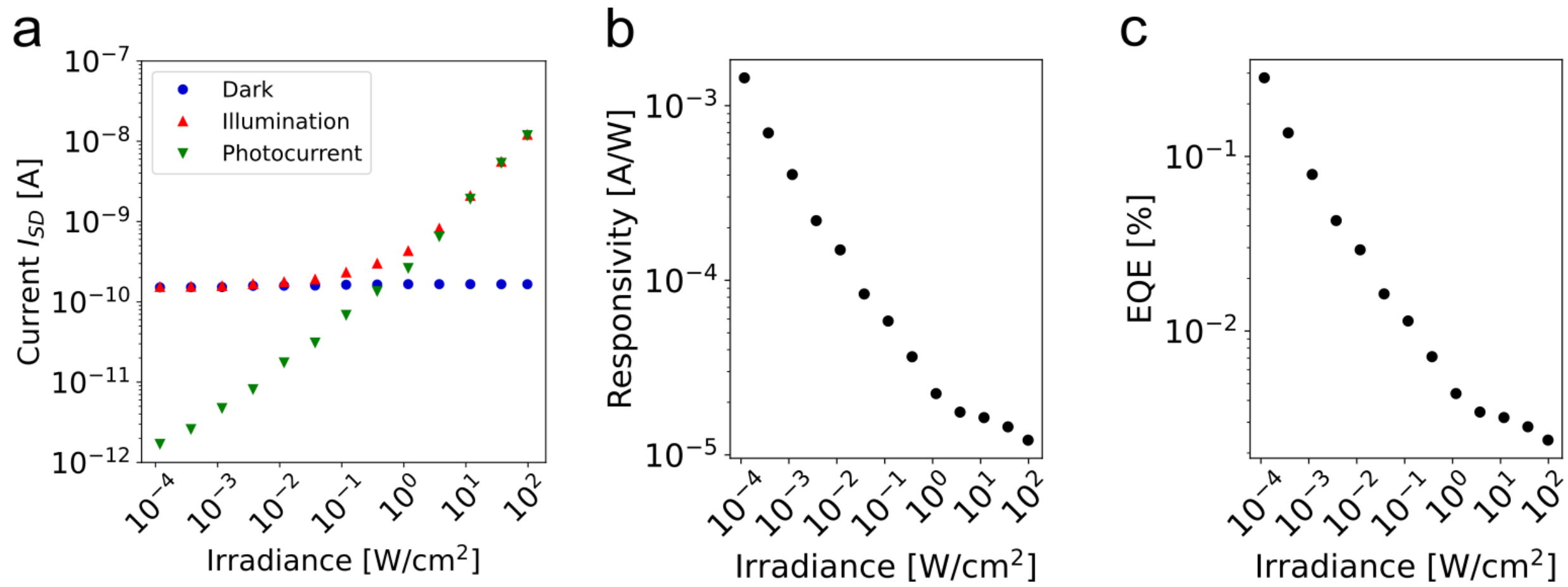

Figure S6. Results of the irradiance-dependent photocurrent measurements depicted in **Fig. S5**. On/Off-ratio and detectivity are depicted in **Fig. 1c** of the main article. **a)** Photocurrents are determined by $I_{Photo} = I_{Illum} - I_{Dark}$. **b)** Irradiance-dependent responsivity. **c)** Irradiance-dependent external quantum efficiency.

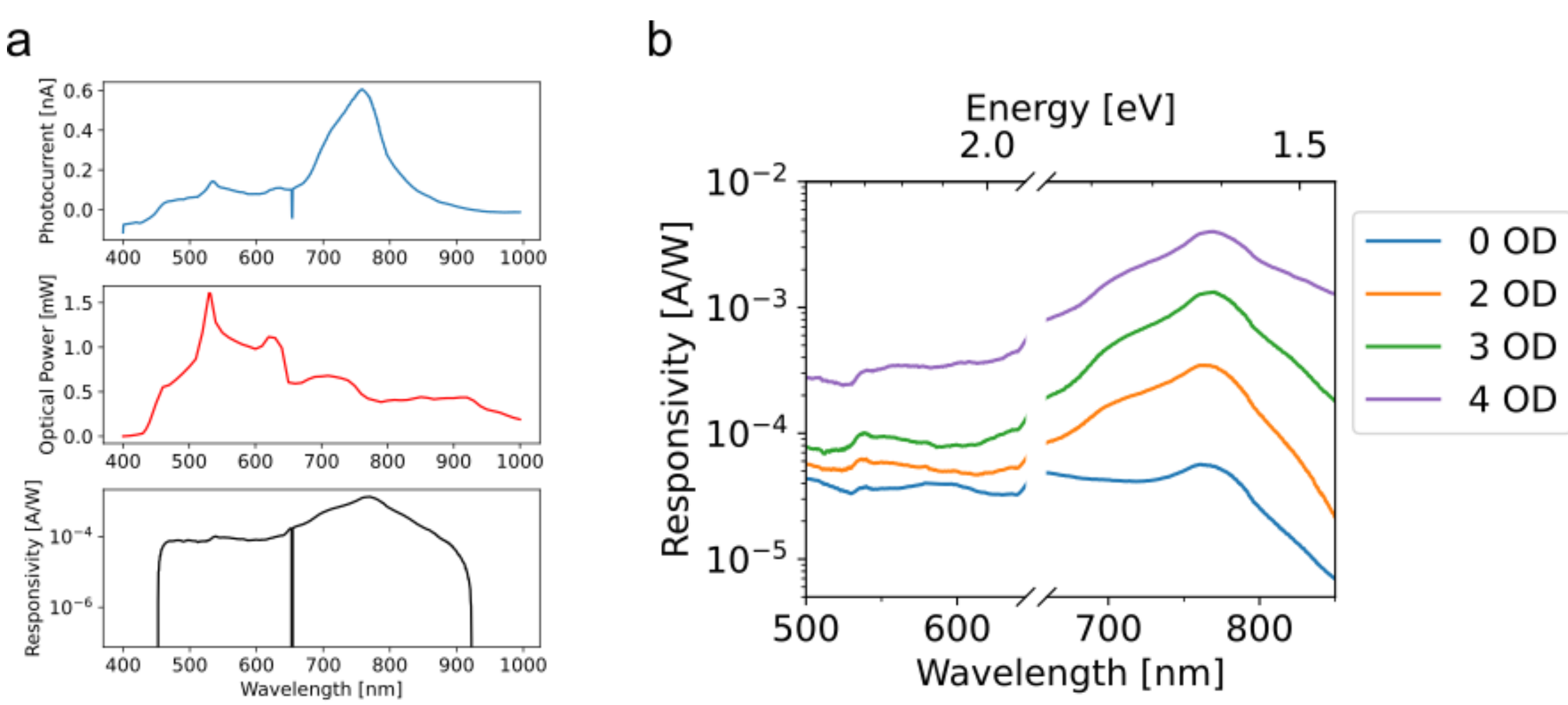

Figure S7. Responsivity spectra of one exemplary device under a bias voltage of 1 V. The channel dimensions are 20 µm × 1 mm. The light was gradually attenuated to demonstrate the intensity-dependence of the responsivity features. **a)** Example for the calculation of responsivities from photocurrents through normalization to the wavelength-dependent optical power. The procedure

is demonstrated for $OD = 3$. The dark current was determined to be 0.58 nA and was subtracted from the currents under illumination. **b)** Responsivity spectra for various laser attenuation values under 1 V bias voltage. The break in the wavelength axis around 650 nm is due to a filter-related artifact.

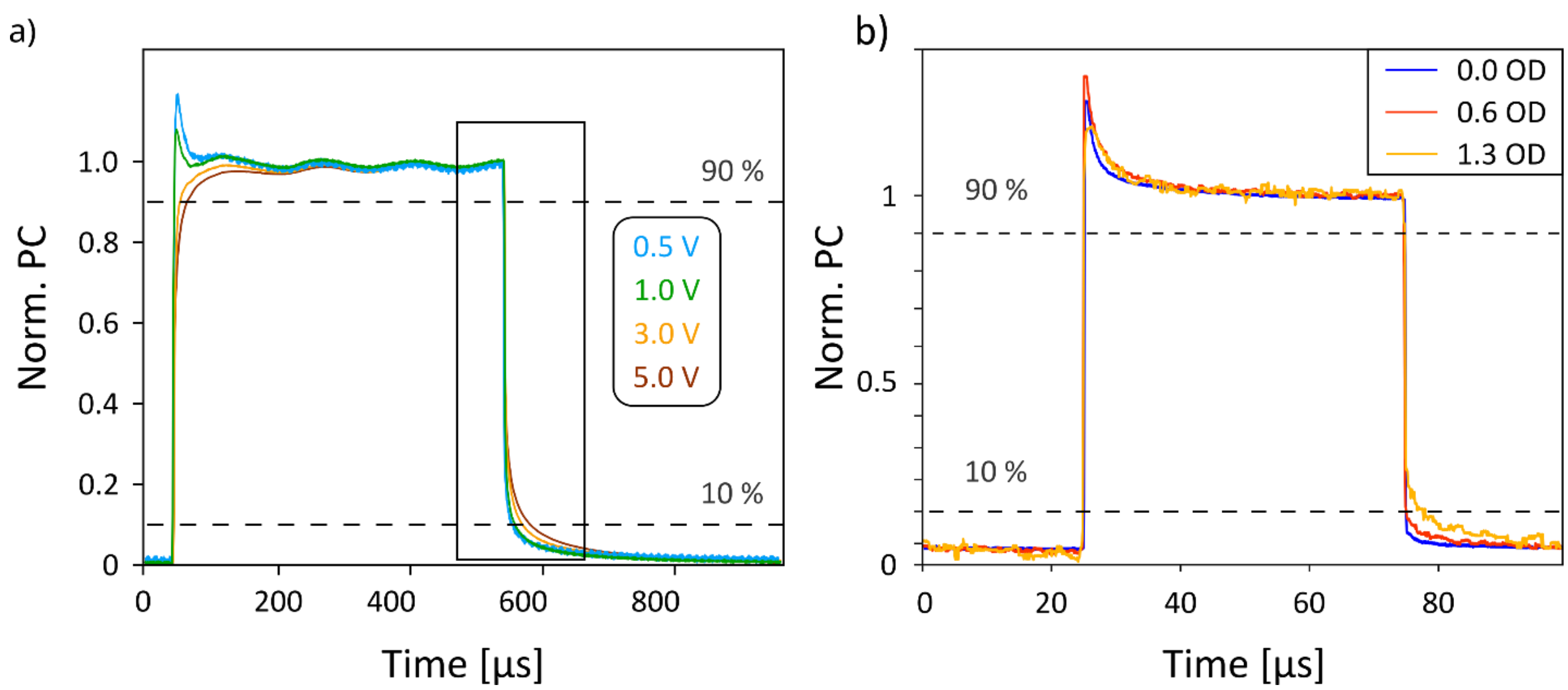

Figure S8. Time-resolved square pulse photoresponse under varying parameters. **a)** Bias-dependent fall time measurements, shown in detail in **Fig. 2a**. Experiments were conducted under 635 nm CW illumination with an irradiance of approximately 2×10^{-3} W/cm^2 (28 µW focused on approx. 1.5 mm spot diameter), with a square pulse repetition rate 1 kHz. The rising flanks exhibit overshooting behavior, a characteristic artifact commonly observed at this repetition frequency in our system. The channel dimensions are 25 µm × 1 mm. **b)** Irradiance-dependent square pulse photoresponse under 3 V bias voltage and 635 nm CW illumination. Here, we show exemplary data for three OD values of 0, 0.6 and 1.3, which correspond to irradiances of 8×10^4 W/cm^2, 2×10^4 W/cm^2 and 4×10^3 W/cm^2, respectively (665 µW focused on approx. 1 µm spot diameter).

The summarized results of irradiance-dependent rise- and fall times are shown in **Fig. 2b**. The channel dimensions are 40 µm in length and 1 mm width.

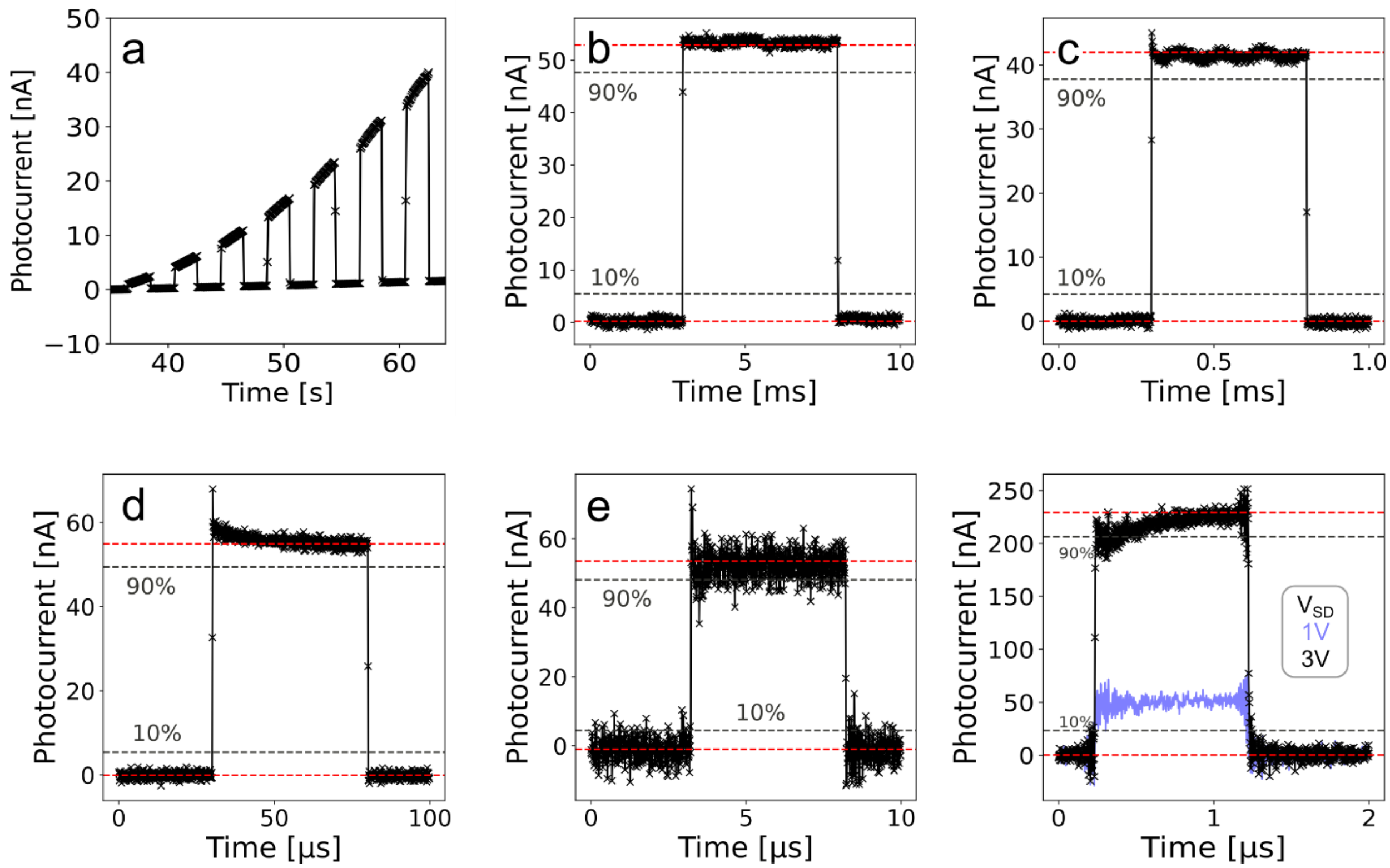

Figure S9. Time-resolved square-pulse measurements for an exemplary device with 50 µm channel length and 1 mm channel width. The applied 635 nm irradiance was approx. 8×10^4 W/cm^2 (665 µW focused on approx. 1 µm spot diameter). **a)** Time-resolved photocurrent measurements under a bias gradient from 0 V to 3 V, recorded with a Keithley 2634B source meter. The temporal resolution of 70 ms is insufficient to resolve the falling and rising flanks. **b-f)** Square pulse measurements utilizing an UHFLI lock-in amplifier. The laser repetition frequencies for b-f) are 100 Hz, 1 kHz, 10 kHz, 100 kHz and 500 kHz, respectively. For the highest repetition rate of 500 kHz (f), the bias voltage was increased from 1 V to 3 V for an improved signal-to noise ratio. The results of the measurements are summarized in **Table S1**.

Table S1. Summarized results of the square-pulse measurements presented in **Fig. S9b-f**. The temporal resolution is provided as temporal distance between two data points. Only at a 500 kHz repetition rate, the rising and falling flanks are resolved with >2 data points, indicating a limitation by the setup for repetition rates <500 kHz. Since the rising and falling flanks demonstrate prompt switching throughout repetition frequencies from 100 Hz to 500 kHz with no significant decrease of the photocurrent, we conclude that no slow signal components are neglected

**Laser Repetition Rate**	**Temporal Resolution**	**Rise Time**	**Fall Time**	**Bias**	**Photocurrent**
100 Hz	9.77 µs	9.77 µs	19.5 µs	1 V	52.7 nA
1 kHz	0.977 µs	1.95 µs	2.93 µs	1 V	42.0 nA
10 kHz	97.7 ns	195 ns	195 ns	1 V	55.0 nA
100 kHz	9.77 ns	19.5 ns	19.5 ns	1 V	54.5 nA
500 kHz	1.95 ns	13.7 ns	15.6 ns	1 V	53.0 nA
500 kHz	1.95 ns	9.77 ns	9.77 ns	3 V	229.0 nA

Table S2. Results of the irradiance-dependent response time measurements summarized in **Fig. 2b**. The sample was illuminated by a 635 nm spare pulse laser at a 10 kHz repetition rate (98 ns temporal resolution). The device with 40 µm channel length and 1 mm channel width was operated under 3 V bias. For additional measurements at 100 kHz, see **Table S3**

**OD**	**Irradiance [W/cm²]**	**Power [µW]**	**Photocurrent [nA]**	**Responsivity [A/W]**	**Rise Time [ns]**	**Fall Time [ns]**
0	8.5×10^{4}	665	25	3.8×10^{-5}	98	98
0.3	4.2×10^{4}	333	14.7	4.4×10^{-5}	98	195
0.6	2.1×10^{4}	167	8.81	5.3×10^{-5}	98	488
0.9	1.1×10^{4}	132	5.28	4.0×10^{-5}	98	1170
1.3	4.3×10^{3}	33.3	2.64	7.9×10^{-5}	195	2640
1.8	1.3×10^{3}	10.5	2.68	2.5×10^{-4}	195	4000
2.3	4.3×10^{2}	3.3	1.04	3.1×10^{-4}	1460	6450

Table S3. Results of the irradiance-dependent response time measurements summarized in the inset of **Fig. 2b**. The sample was illuminated by a 635 nm spare pulse laser at a 100 kHz repetition rate (9.8 ns temporal resolution), since the temporal resolution limit at 10 kHz (98 ns) was reached for low OD values (cf. **Table S2**)

**OD**	**Irradiance [W/cm²]**	**Power [µW]**	**Photocurrent [nA]**	**Responsivity [A/W]**	**Rise Time [ns]**	**Fall Time [ns]**
0.0	8.5×10^{4}	665	33.8	5.1×10^{-5}	19.5	58.6
0.3	4.2×10^{4}	333	19.0	5.7×10^{-5}	29.3	68.4
0.6	2.1×10^{4}	167	9.13	5.5×10^{-5}	87.9	107

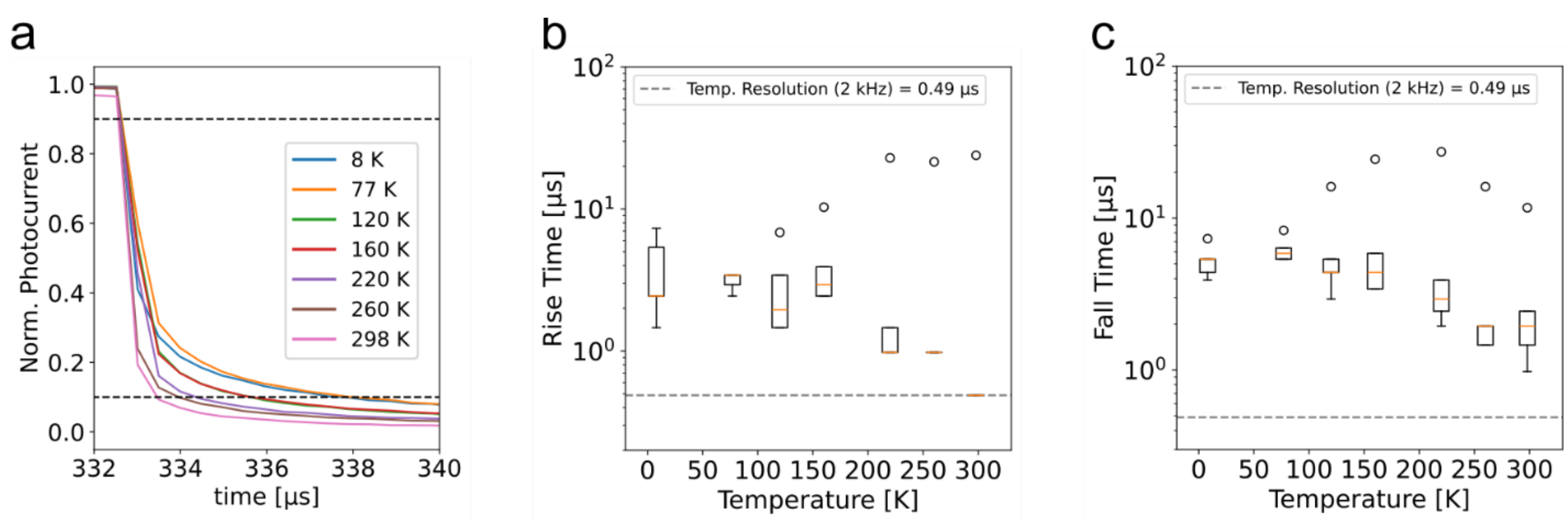

Figure S10. Temperature-dependent response times for five channels with 30 µm length and 1 mm width, cf. **Fig. S1a,b**. The irradiance of the 635 nm illumination was approx. 2×10^{-3} W/cm² (28 µW focused on approx. 1.5 mm spot diameter), with a square pulse repetition rate of 2 kHz. The device was operated under 3 V bias voltage. **a)** Temperature-dependent photocurrent decay for one exemplary channel, demonstrating increasing fall times with decreasing temperature. **b,c)** Boxplot representation of temperature-dependent rise- and fall times, respectively. Except for one channel, the response times decrease with temperature.

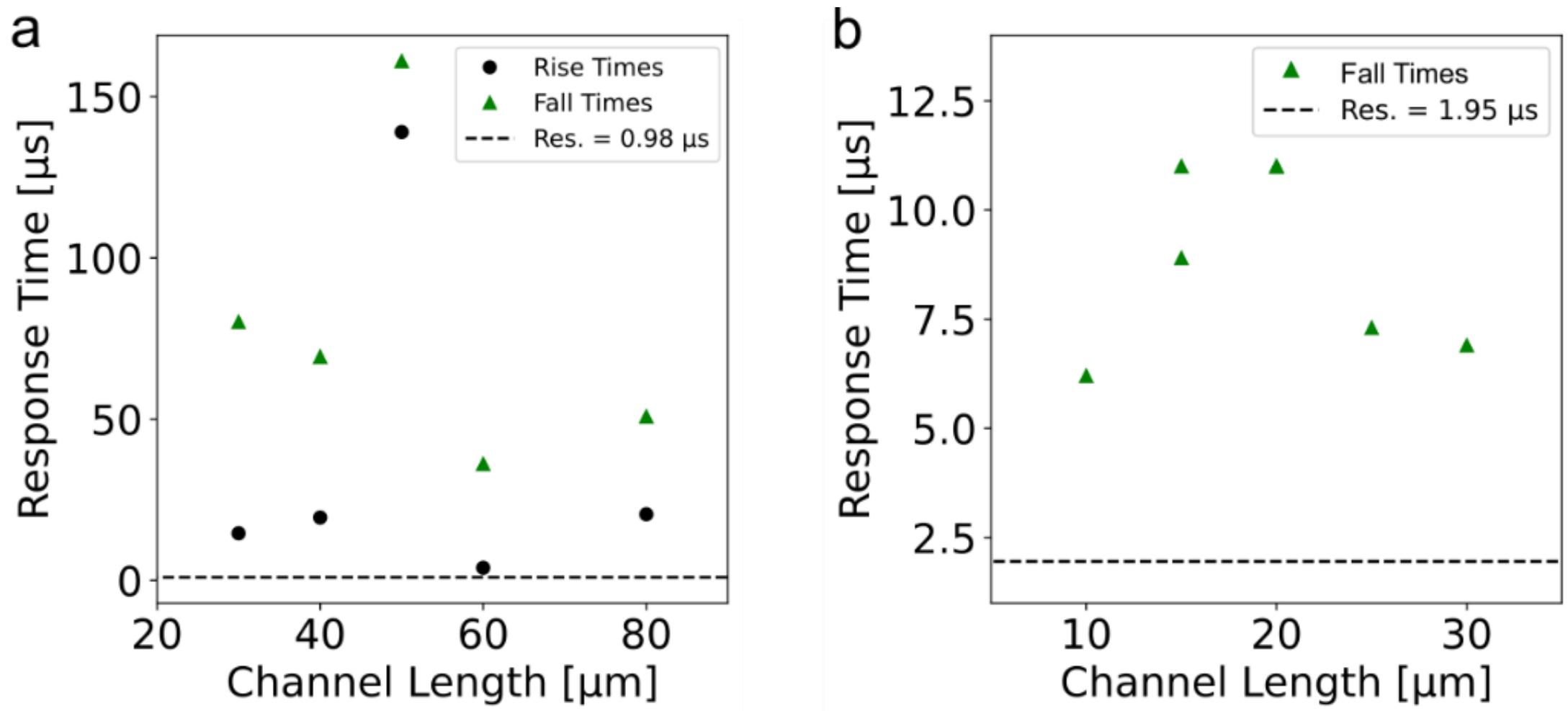

Figure S11. Comparison of response times for different channel lengths across two samples shown in **a)** and **b)**, respectively. The devices were operated under 3 V bias voltage. The channel width was 1 mm throughout all channels (cf. Fig. **S1a,b**). In both samples, no consistent relationship between channel length and response time was observed. We conclude that the variance among individual channels is too large to identify a definitive trend. Measurements were performed under 635 nm illumination with approx. 2×10^{-3} W/cm^2 (28 µW focused on approx. 1.5 mm spot diameter), with a square pulse repetition rate 1 kHz (a) and 500 Hz (b). The instrumental resolutions at the respective repetition rates are shown in dashed lines.

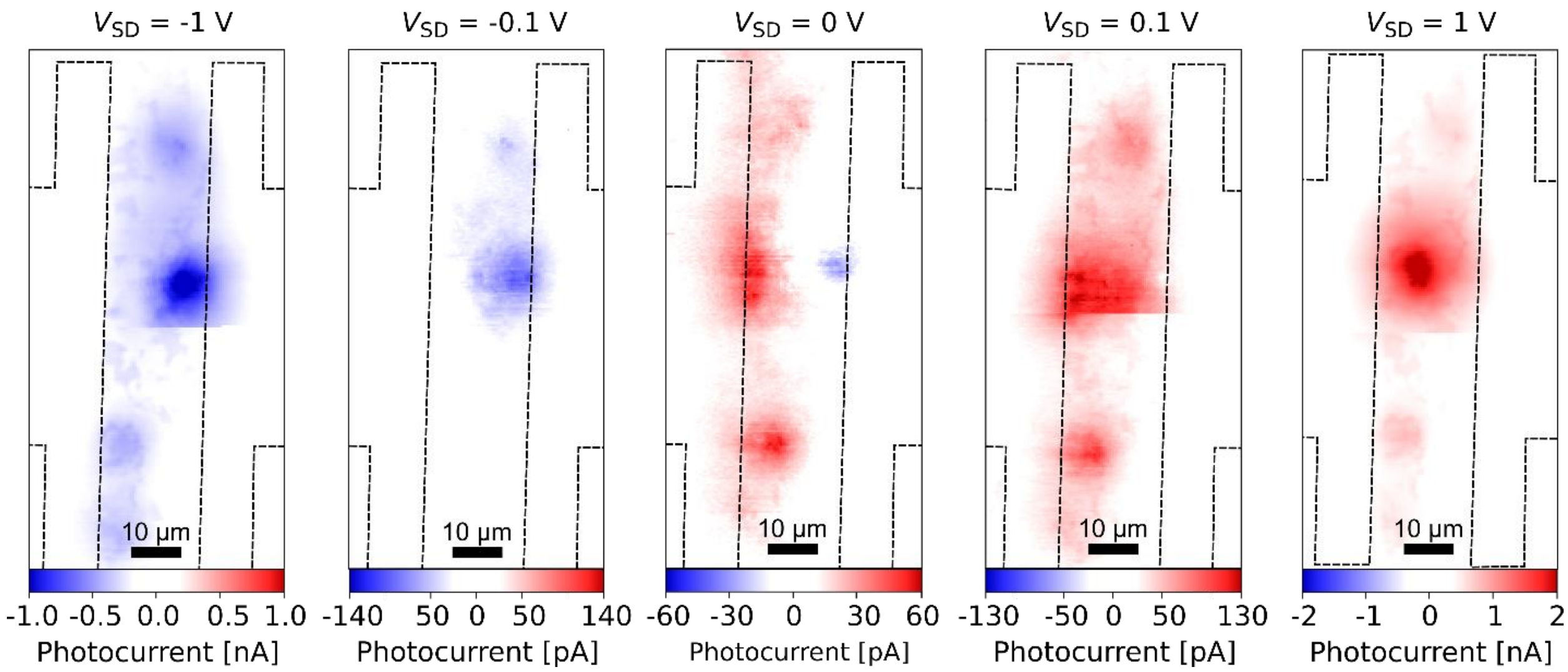

Figure S12. Non-logarithmic representation of the bias-dependent scanning photocurrent micrographs depicted in **Fig. 3**. An optical micrograph of the sample is provided in **Fig. S1c**. Notably, the ranges are non-uniform for different voltages. With increased bias voltage, an expansion of the PC generating zones ("hot spots") is observed. All images are stitched from two scans (lower and upper part).

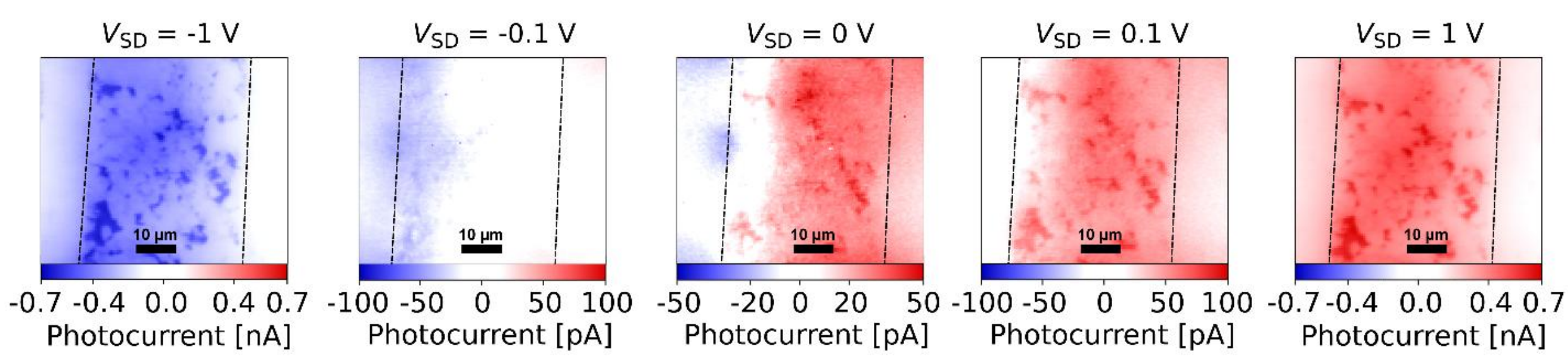

Figure S13 Bias-dependent scanning photocurrent micrographs for a 40 µm wide channel. An optical micrograph of the sample is provided in **Fig. S1d**. Notably, the ranges are non-uniform for different voltages. With increased bias voltage, an expansion of the PC-generation is observed. We attribute the lower currents compared to **Fig. S12** to an increased channel length, resulting in higher device resistance. Notably, increased photocurrent generation is observed at gaps in the WSe_2 film (cf. **Fig S1d**), which seems counterintuitive. We suggest that the laser, which is

transmitted through the blank substrate, may be scattered by the underlying PCB gold surface, broadly illuminating the sample and thereby generating photocurrents. We therefore consider those spots systemic artefacts rather than photocurrent generating hotspots.

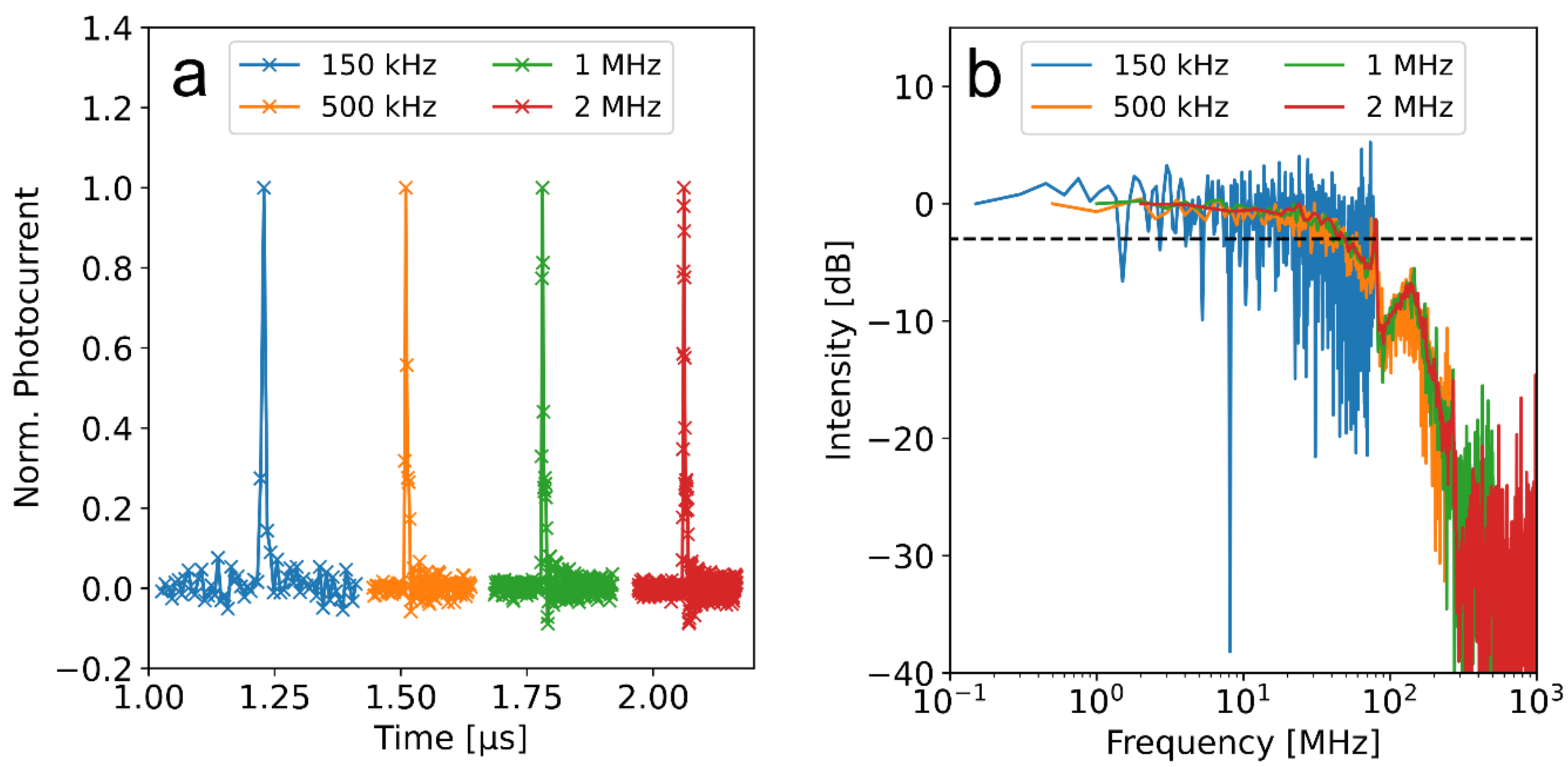

Figure S14. Time-resolved impulse measurements for an exemplary device with 20 μm channel length and 1 mm channel width. Illumination was performed with a 532 nm IP laser (average irradiance ≈2×10^3 W/cm^2). The bias is 1V. For a tabular representation of the results, see **Table S4**. **a)** Relaxation of the impulse response is demonstrated for repetition rates between 150 kHz and 2 MHz. With increasing repetition rate, the temporal resolution of the signal is enhanced. **b)** Power spectra obtained by FFT of the data presented in a). With increasing repetition rate and increased temporal resolution, the noise in the power spectra is reduced, resulting in a more accurate determination of the bandwidth.

Table S4. Summarized results of the impulse measurements presented in **Fig. S14.** The temporal resolution is provided as temporal distance between two data points

Laser Repetition Rate	Temporal Resolution	Photocurrent	Fall Time	Bandwidth
150 kHz	6.5 ns	234 nA	26 ns	1.4 MHz
500 kHz	2 ns	388 nA	9.8 ns	24 MHz
1 MHz	1 ns	377 nA	7.8 ns	41 MHz
2 MHz	0.5 ns	366 nA	7.8 ns	44 MHz

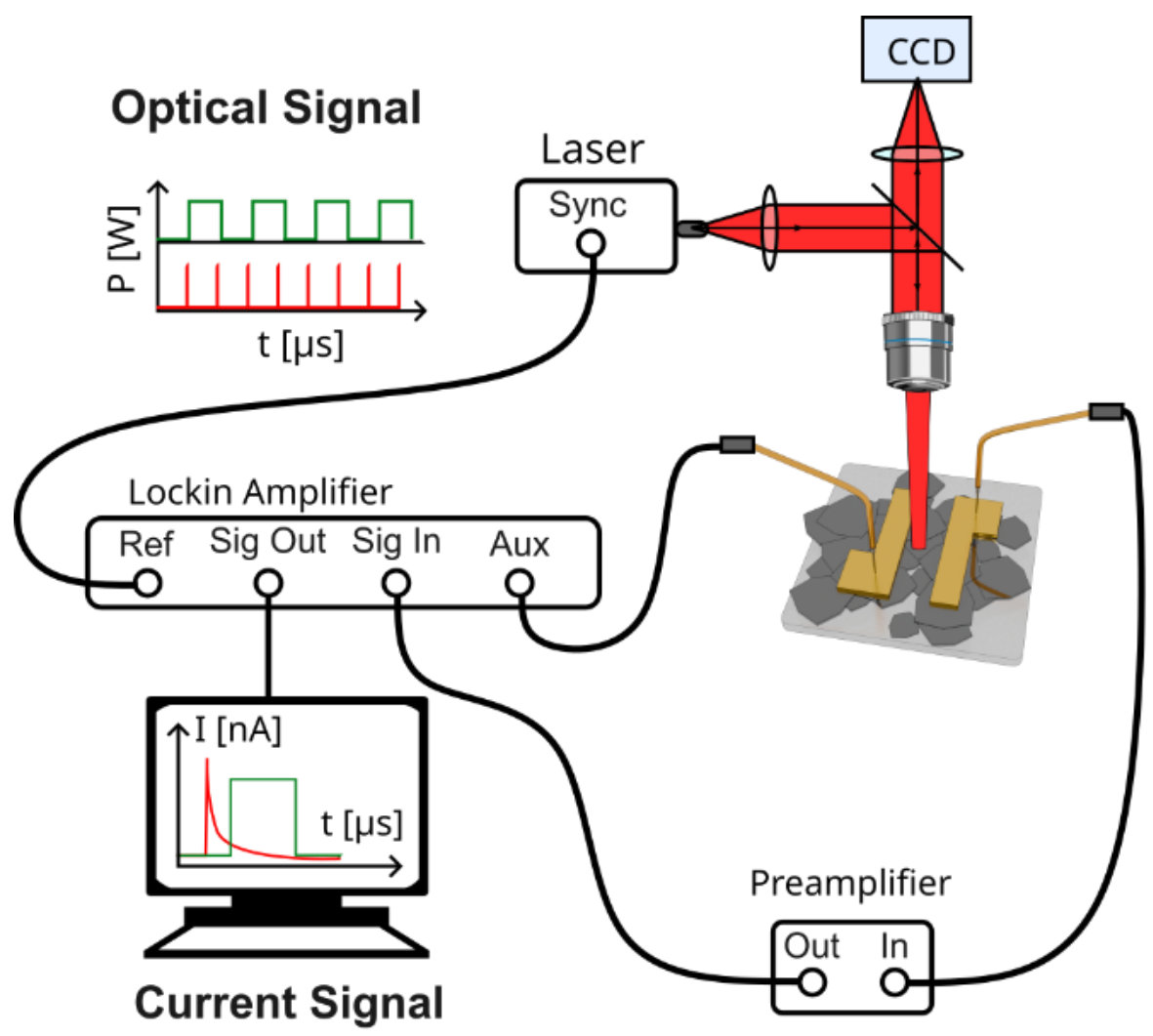

Figure S15. Experimental setup for time-resolved photocurrent detection under focused laser irradiance. For a more detailed description of the setup, the reader is referred to the "Methods - Optoelectronic characterization" section above.

Table S5. Electrical and optical components used in the confocal setup for high-speed photodetection presented in **Figure S15**, as well as their speed-limiting specifications

Component	Model	Speed-limiting Specification
Square Pulse Laser	PicoQuant FSL500	rise/fall time < 3 ns
Impulse Laser	NKT SuperK FIU-15 PP	pulse width ≈ 50 ps
Contact tips	W-tips (44-8000-S-NA)	3 GHz bandwidth
Cables	60 cm RG178 B/U cables	3 GHz bandwidth
Preamplifier	FEMTO DHPCA-100	200 MHz, 1.8 ns rise time (at 1e2 V/A)<br>80 MHz, 4.4 ns rise time (at 1e3 V/A)
Lock-in amplifier	Zürich Instruments UHF Lock-In	600 MHz bandwidth

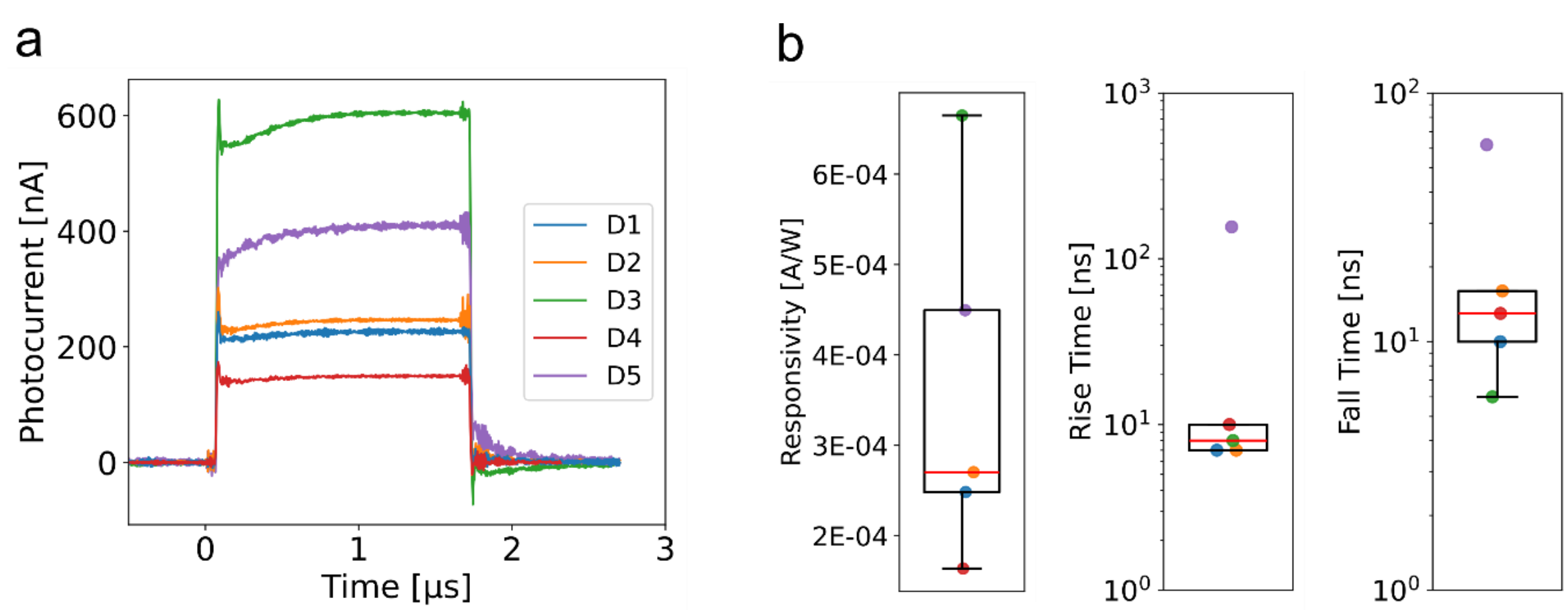

Figure S16. Time-resolved photoresponse of five devices (20 µm channel length, 1 mm channel width) towards a square pulse modulated 635 nm CW laser (power = 0.9 mW, irradiance ≈ 1×10^5 W/cm^2) operated at 3 V bias with a 300 kHz repetition rate. **a)** Time-resolved

photocurrent representation **b)** Boxplot representation of the responsivity, rise time, and fall time obtained from the measurements shown in a).

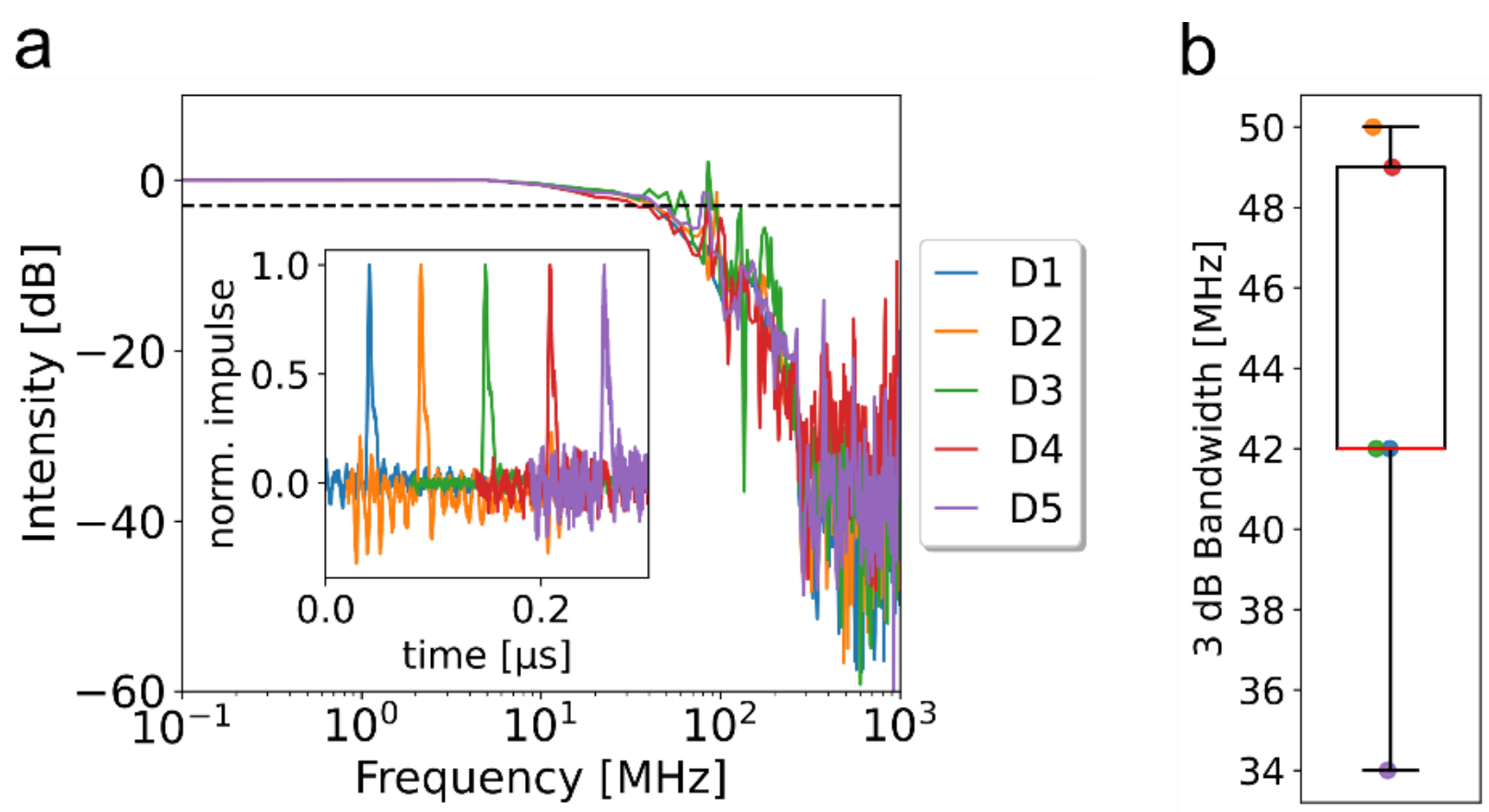

Figure S17. Time-resolved photoresponse of five devices (20 μm channel length, 1 mm channel width) towards a 635 nm impulse laser (average power ≈ 1×10^{-5} W, irradiance ≈ 1×10^{3} W/cm^2) operated at 3 V bias with a 5 MHz repetition rate. **a)** Power spectra of the five devices, visualizing the 3 dB cutoff frequency. Inset: Time-resolved photocurrent representation (offset for improved clarity). **b)** Boxplot representation of the 3 dB bandwidth obtained from the measurements shown in a).

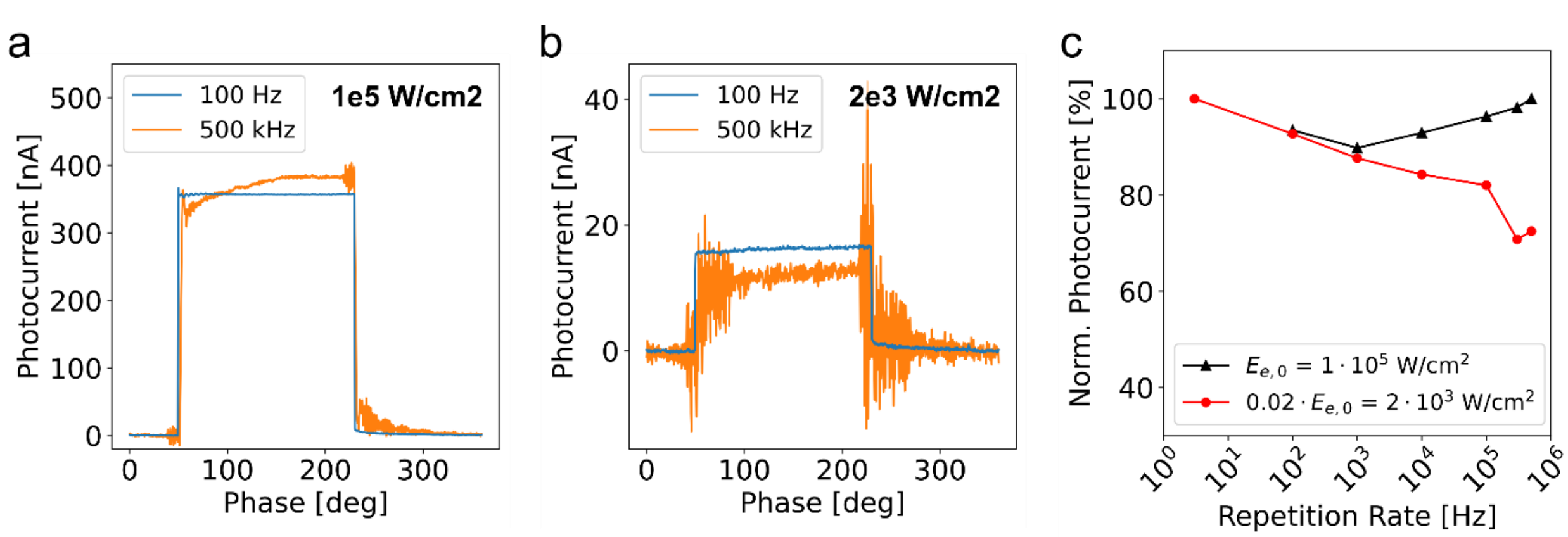

Figure S18. Quantitative investigation of current attenuation with increasing pulse repetition rate, recorded for different irradiances. A device (20 µm channel length, 1 mm channel width) was excited with a square pulse modulated 635 nm CW laser towards a square pulse modulated 635 nm CW under 10 V bias. **a)** Under high irradiance (1×10^5 W/cm^2), no attenuation is observed when the repetition rate is increased from 100 Hz to 300 kHz. **b)** When the irradiance is reduced to 2×10^2 W/cm^2, the device speed decreases and steady-state conditions are no longer reached. Therefore, a signal attenuation is observed with increasing repetition rate. **c)** Normalized signal attenuation for a set of repetition rates ranging from 3 Hz to 500 kHz, measured under higher and lower irradiance. Even though steady-state conditions are not reached for the lower irradiance at high repetition rates, approximately 70% of the initial photocurrent are still detected at 500 kHz.

Table S6. Literature review of responsivities and response times for TMDC-based photodetectors. While we focus on scalable TMDC fabrication, approaches like roll-to roll exfoliation (RR), electrodeposition (ED), liquid phase exfoliation (LE), chemical vapor deposition (CVD), metal-organic vapor phase epitaxy (MOVPE), pulsed laser deposition (PLD), two-step thermolysis (TT) and sputtering (SP), the fastest results achieved through mechanical exfoliation (ME) are provided for comparison. A graphical representation of this review is given in **Fig. 5**

**Method**	**Material**	**WL [nm]**	**Responsivity [A/W]**	**Rise Time [s]**	**Fall Time [s]**	**Bandwidth [MHz]**	**Ref.**
RR	Bulk WSe_2	635, 636	3.4×10^{-4}	10×10^{-9}	10×10^{-9}	54	This Work
ED	Bulk MoS_2	532	0.9×10^{-3}	≈1	≈1	-	[4]
LE	Few-layer MoS_2	632	128×10^{-3}	80.31×10^{-6}	60.65×10^{-6}	-	[5]
LE	Few-layered WSe_2	AM1.5D	$\approx1\times10^{-5}$	<1	<1	-	[6]
LE	Bulk WSe_2	Pink	3.65×10^{-6}	453×10^{-3}	504×10^{-3}	-	[7]
CVD	Few-layer MoS_2	532	20×10^{-3}	12	19	-	[8]
CVD	Monolayer MoS_2	610	$\approx4\times10^{-3}$	1.03	1.03	-	[9]
CVD	Monolayer WS_2	532	4500	$<2\times10^{-3}$	$<2\times10^{-3}$	-	[10]
CVD	Monolayer WS_2	-	2.5×10^{4}	58×10^{-6}	-	-	[11]
CVD	Few-layer $MoSe_2$	638	859×10^{-3}	40×10^{-3}	38×10^{-3}	-	[12]
CVD	Bilayered WS_2	220	0.92	-	100	-	[13]
CVD	Monolayer MoS_2	640	610	3.2	7.0	-	[14]

CVD	Monolayer MoS2	520	2×10^{-2}	-	1.7	-	15
CVD	Monolayer WS2	532	0.2×10^{-6}	$<4.5\times10^{-3}$	$<4.5\times10^{-3}$	-	16
CVD	Monolayer WS2	532	0.52×10^{-3}	$<560\times10^{-6}$	$<560\times10^{-6}$	-	17
CVD	Monolayer MoS2	450	40	1.29	2.23	-	18
CVD	Few-layer WS2	VIS	92×10^{-6}	5.3×10^{-3}	5.3×10^{-3}	-	19
MOVPE	Few-layer MoS2	White	540	0.5	1.15	-	20
PLD	Bulk WSe2	635	0.92	0.9	2	-	21
PLD	Bulk WS2	635	0.51	4.1	4.4	-	22
PLD	Monolayer MoS2	650	≈0.75	96×10^{-3}	96×10^{-3}	-	23
TT	Few-layer MoS2	532	0.57	70×10^{-6}	110×10^{-6}	-	24
SP	Bulk MoS2	808	300×10^{-3}	3×10^{-6}	40×10^{-6}	-	25
SP	Amorphous MoS2	1550	3.15×10^{-3}	10×10^{-3}	16×10^{-3}	-	26
ME	Bilayered WSe2	635	6.5×10^{-3}	29×10^{-9}	19×10^{-9}	-	27
ME	Few-layered WSe2	636	0.05	-	-	230	27
ME	Bulk WSe2	450	0.17	8×10^{-9}	20×10^{-9}	22	28
ME	Bulk MoTe2	980	0.33	22×10^{-9}	24×10^{-9}	-	28
ME	Bulk WSe2	450	0.1	264×10^{-9}	552×10^{-9}	1.9	29
ME	Few-layer WSe2	635	1.3×10^{-4}	8×10^{-9}	7×10^{-9}	63	30

References

(1) Sozen, Y.; Riquelme, J. J.; Xie, Y.; Munuera, C.; Castellanos-Gomez, A. High-Throughput Mechanical Exfoliation for Low-Cost Production of van Der Waals Nanosheets. *Small Methods* **2023**, *7* (10), e2300326. https://doi.org/10.1002/smtd.202300326.

(2) Sozen, Y.; Pucher, T.; Kesavan, B. P.; Jimenez-Arevalo, N.; Hernandez-Ruiz, J.; Sofer, Z.; Munuera, C.; Riquelme, J. J.; Castellanos-Gomez, A. Wafer-Scale Films of Two-Dimensional Materials via Roll-to-Roll Mechanical Exfoliation. arXiv 2025, Access: 2025-11-24. https://doi.org/10.48550/ARXIV.2511.06960.

(3) Mao, D.; She, X.; Du, B.; Yang, D.; Zhang, W.; Song, K.; Cui, X.; Jiang, B.; Peng, T.; Zhao, J. Erbium-Doped Fiber Laser Passively Mode Locked with Few-Layer WSe2/MoSe2 Nanosheets. *Sci. Rep.* **2016**, *6* (1), 23583. https://doi.org/10.1038/srep23583.

(4) Abdelazim, N. M.; Noori, Y. J.; Thomas, S.; Greenacre, V. K.; Han, Y.; Smith, D. E.; Piana, G.; Zhelev, N.; Hector, A. L.; Beanland, R.; Reid, G.; Bartlett, P. N.; Groot, C. H. Lateral Growth of MoS 2 2D Material Semiconductors Over an Insulator Via Electrodeposition. *Adv. Electron. Mater.* **2021**, *7* (9). https://doi.org/10.1002/aelm.202100419.

(5) Abdullah Ripain, A. H.; Zulkifli, N. A. A.; Tan, C. L.; Abd Majid, W. H.; Zakaria, R. Contributions of Symmetric Metal Contacts on Liquid Exfoliation 2D-MoS2 Flakes Based MSM Photodetector by Spray Pyrolysis: A CVD-Free Technique. *Opt. Quantum Electron.* **2022**, *54* (12). https://doi.org/10.1007/s11082-022-04253-y.

(6) Liu, S.; Carey, T.; Munuera, J.; Synnatschke, K.; Kaur, H.; Coleman, E.; Doolan, L.; Coleman, J. N. Solution-Processed Heterojunction Photodiodes Based on WSe_2 Nanosheet Networks. *Small* **2025**, *21* (28), 2304735. https://doi.org/10.1002/smll.202304735.

(7) Chauhan, B. L.; Bhakhar, S. A.; Pataniya, P. M.; Gupta, S. U.; Solanki, G. K.; Pathak, V. M.; Patel, V. Liquid-Phase Exfoliation of WSe2 Nanosheets for ITO/WSe2 Photodetector. *J. Mater. Sci. Mater. Electron.* **2022**, *33* (13), 10314–10322. https://doi.org/10.1007/s10854-022-08019-w.

(8) Yu, F.; Hu, M.; Kang, F.; Lv, R. Flexible Photodetector Based on Large-Area Few-Layer MoS2. *Prog. Nat. Sci. Mater. Int.* **2018**, *28* (5), 563–568. https://doi.org/10.1016/j.pnsc.2018.08.007.

(9) Aggarwal, P.; Kaushik, S.; Bisht, P.; Sharma, M.; Singh, A.; Mehta, B. R.; Singh, R. Centimeter-Scale Synthesis of Monolayer WS 2 Using Single-Zone Atmospheric-Pressure Chemical Vapor Deposition: A Detailed Study of Parametric Dependence, Growth Mechanism, and Photodetector Properties. *Cryst. Growth Des.* **2022**, *22* (5), 3206–3217. https://doi.org/10.1021/acs.cgd.2c00049.

(10) Aji, A. S.; Solís-Fernández, P.; Ji, H. G.; Fukuda, K.; Ago, H. High Mobility WS 2 Transistors Realized by Multilayer Graphene Electrodes and Application to High Responsivity Flexible Photodetectors. *Adv. Funct. Mater.* **2017**, *27* (47). https://doi.org/10.1002/adfm.201703448.

(11) Chen, Y.-S.; Chiu, S.-K.; Tsai, D.-L.; Liu, C.-Y.; Ting, H.-A.; Yao, Y.-C.; Son, H.; Haider, G.; Kalbáč, M.; Ting, C.-C.; Chen, Y.-F.; Hofmann, M.; Hsieh, Y.-P. Mediator-Assisted Synthesis

of WS2 with Ultrahigh-Optoelectronic Performance at Multi-Wafer Scale. *Npj 2D Mater. Appl.* **2022**, *6* (1). https://doi.org/10.1038/s41699-022-00329-1.

(12) Dai, T.; Liu, Y.; Liu, X.; Xie, D.; Li, Y. High Performance Photodetectors Constructed on Atomically Thin Few-Layer MoSe2 Synthesized Using Atomic Layer Deposition and a Chemical Vapor Deposition Chamber. *J. Alloys Compd.* **2019**, *785*, 951–957. https://doi.org/10.1016/j.jallcom.2019.01.206.

(13) Hussain, S.; Khan, M. F.; Shehzad, M. A.; Vikraman, D.; Iqbal, M. Z.; Choi, D.-C.; Song, W.; An, K.-S.; Seo, Y.; Eom, J.; Lee, W.-G.; Jung, J. Layer-Modulated, Wafer Scale and Continuous Ultra-Thin WS 2 Films Grown by RF Sputtering via Post-Deposition Annealing. *J. Mater. Chem. C* **2016**, *4* (33), 7846–7852. https://doi.org/10.1039/C6TC01954D.

(14) Hwa, Y.; Chee, S.-S. Improvement of Visible Photodetection of Chemical Vapor Deposition-Grown MoS2 Devices via Graphene/Au Contacts. *Sensors* **2022**, *22* (24). https://doi.org/10.3390/s22249687.

(15) Kim, T.-Y.; Ha, J.; Cho, K.; Pak, J.; Seo, J.; Park, J.; Kim, J.-K.; Chung, S.; Hong, Y.; Lee, T. Transparent Large-Area MoS2 Phototransistors with Inkjet-Printed Components on Flexible Platforms. *ACS Nano* **2017**, *11* (10), 10273–10280. https://doi.org/10.1021/acsnano.7b04893.

(16) Lan, C.; Li, C.; Yin, Y.; Liu, Y. Large-Area Synthesis of Monolayer WS_2 and Its Ambient-Sensitive Photo-Detecting Performance. *Nanoscale* **2015**, *7* (14), 5974–5980. https://doi.org/10.1039/C5NR01205H.

(17) Lan, C.; Zhou, Z.; Zhou, Z.; Li, C.; Shu, L.; Shen, L.; Li, D.; Dong, R.; Yip, S.; Ho, J. C. Wafer-Scale Synthesis of Monolayer WS2 for High-Performance Flexible Photodetectors by

Enhanced Chemical Vapor Deposition. *Nano Res.* **2018**, *11* (6), 3371–3384. https://doi.org/10.1007/s12274-017-1941-4.

(18) Pak, S.; Kim, S.; Lim, J.; Kim, T.; Park, K.-H.; Cha, S. Direct CVD Synthesis of MoS 2 Monolayers on Glass by Carbothermal Reduction. *J. Phys. Chem. C* **2023**, *127* (9), 4689–4695. https://doi.org/10.1021/acs.jpcc.2c07542.

(19) Perea-López, N.; Elías, A. L.; Berkdemir, A.; Castro-Beltran, A.; Gutiérrez, H. R.; Feng, S.; Lv, R.; Hayashi, T.; López-Urías, F.; Ghosh, S.; Muchharla, B.; Talapatra, S.; Terrones, H.; Terrones, M. Photosensor Device Based on Few-Layered WS 2 Films. *Adv. Funct. Mater.* **2013**, *23* (44), 5511–5517. https://doi.org/10.1002/adfm.201300760.

(20) Schneider, D. S.; Grundmann, A.; Bablich, A.; Passi, V.; Kataria, S.; Kalisch, H.; Heuken, M.; Vescan, A.; Neumaier, D.; Lemme, M. C. Highly Responsive Flexible Photodetectors Based on MOVPE Grown Uniform Few-Layer MoS 2. *ACS Photonics* **2020**, *7* (6), 1388–1395. https://doi.org/10.1021/acsphotonics.0c00361.

(21) Zheng, Z.; Zhang, T.; Yao, J.; Zhang, Y.; Xu, J.; Yang, G. Flexible, Transparent and Ultra-Broadband Photodetector Based on Large-Area WSe2 Film for Wearable Devices. *Nanotechnology* **2016**, *27* (22), 225501. https://doi.org/10.1088/0957-4484/27/22/225501.

(22) Yao, J. D.; Zheng, Z. Q.; Shao, J. M.; Yang, G. W. Stable, Highly-Responsive and Broadband Photodetection Based on Large-Area Multilayered WS2 Films Grown by Pulsed-Laser Deposition. *Nanoscale* **2015**, *7* (36), 14974–14981. https://doi.org/10.1039/C5NR03361F.

(23) Jiao, L.; Jie, W.; Yang, Z.; Wang, Y.; Chen, Z.; Zhang, X.; Tang, W.; Wu, Z.; Hao, J. Layer-Dependent Photoresponse of 2D MoS 2 Films Prepared by Pulsed Laser Deposition. *J. Mater. Chem. C* **2019**, *7* (9), 2522–2529. https://doi.org/10.1039/C8TC04612C.

(24) Tsai, D.-S.; Liu, K.-K.; Lien, D.-H.; Tsai, M.-L.; Kang, C.-F.; Lin, C.-A.; Li, L.-J.; He, J.-H. Few-Layer MoS2 with High Broadband Photogain and Fast Optical Switching for Use in Harsh Environments. *ACS Nano* **2013**, *7* (5), 3905–3911. https://doi.org/10.1021/nn305301b.

(25) Wang, L.; Jie, J.; Shao, Z.; Zhang, Q.; Zhang, X.; Wang, Y.; Sun, Z.; Lee, S. MoS 2 /Si Heterojunction with Vertically Standing Layered Structure for Ultrafast, High-Detectivity, Self-Driven Visible–Near Infrared Photodetectors. *Adv. Funct. Mater.* **2015**, *25* (19), 2910–2919. https://doi.org/10.1002/adfm.201500216.

(26) Huang, Z.; Zhang, T.; Liu, J.; Zhang, L.; Jin, Y.; Wang, J.; Jiang, K.; Fan, S.; Li, Q. Amorphous MoS 2 Photodetector with Ultra-Broadband Response. *ACS Appl. Electron. Mater.* **2019**, *1* (7), 1314–1321. https://doi.org/10.1021/acsaelm.9b00247.

(27) Strauß, F.; Kohlschreiber, P.; Keck, J.; Michel, P.; Hiller, J.; Meixner, A. J.; Scheele, M. A Simple 230 MHz Photodetector Based on Exfoliated WSe_2 Multilayers. *RSC Appl. Interfaces.* **2024**, *1* (4), 728–733. https://doi.org/10.1039/D4LF00019F.

(28) Zhang, Y.; Shen, W.; Wu, S.; Tang, W.; Shu, Y.; Ma, K.; Zhang, B.; Zhou, P.; Wang, S. High-Speed Transition-Metal Dichalcogenides Based Schottky Photodiodes for Visible and Infrared Light Communication. *ACS Nano* **2022**, *16* (11), 19187–19198. https://doi.org/10.1021/acsnano.2c08394.

(29) Zhang, Y.; Ma, K.; Zhao, C.; Hong, W.; Nie, C.; Qiu, Z.-J.; Wang, S. An Ultrafast WSe_2 Photodiode Based on a Lateral *p-i-n* Homojunction. *ACS Nano* **2021**, *15* (3), 4405–4415. https://doi.org/10.1021/acsnano.0c08075.

(30) Roberts, W.; Strienz, M.; Bozkurt, A.; Hiller, J. L.; Eberle, M.; Schnepf, A.; Braun, K.; Scheele, M. Direct Laser Writing of Au Top Contacts on WSe_2 for High-Speed Photodetection. *ACS Appl. Nano Mater.* **2025**, *8* (48), 22938–22946. https://doi.org/10.1021/acsanm.5c03363.
